# Brazilian Trichoptera Checklist II

**DOI:** 10.3897/BDJ.2.e1557

**Published:** 2014-10-09

**Authors:** Henrique Paprocki, Diogo França

**Affiliations:** † Pontifícia Universidade Católica de Minas Gerais, Museu de Ciências Naturais, Coleção de Invertebrados. Av. Dom José Gaspar, 290, sala 104, Coração Eucarístico, CEP 30535-901, Belo Horizonte, Minas Gerais, Brazil, Belo Horizonte, Brazil

**Keywords:** Biodiversity, aquatic insects, Trichoptera, caddisflies, inventory, checklist, Brazil

## Abstract

A second assessment of Brazilian Trichoptera species records is presented here. A total of 625 species were recorded for Brazil. This represents an increase of 65.34% new species recorded during the last decade. The Hydropsychidae (124 spp.), followed by the Hydroptilidae (102 spp.) and Polycentropodidae (97 spp.), are the families with the greatest richness recorded for Brazil. The knowledge on Trichoptera biodiversity in Brazil is geographically unequal. The majority of the species is recorded for the southeastern region.

## Introduction

Biodiversity data should be made widely available online to better inform conservation of natural resources and advancement of natural sciences. Meta-analysis in molecular biology and others areas is currently a major source for understanding complex biological systems (Emerson et al. 2011, Vos et al. 2014). Checklists of animal and plants species and their distribution are one of the most useful sources of information in conservation, biodiversity studies, and environmental licensing. Checklists are used by scientists for registering new species records and descriptions, to predict species distribution, to compare environments, and to monitor environmental impacts. As the climate changes, biodiversity data will be very important to understand the adaptation of biological communities to these changes. Conservationists can better predict impact outcomes and propose better conservation plans when checklists are available (Fonseca and Benson 2003).

The first Brazilian Trichoptera checklist was published by Paprocki et al. 2004 and registered 378 species. At that time, the number of people working on Brazilian caddisfly taxonomy could be counted in one hand and almost all of them resided outside of Brazil (e.g. Blahnik, R.J., Flint, O.S., Holzenthal, R.W.) Currently, the number of Trichoptera taxonomists with a Ph.D working and residing in Brazil has increased, but is still low (e.g. Calor, A.R., Dumas, L.L., Nessimian, J.L., Pes, A.M.O., Santos, A.P.M, Takiya, D.M.). More comprehensive checklists with detailed distribution of species would be even more useful in biodiversity analysis and decision making.

The second checklist of Brazilian Trichoptera is presented here with the addition of a historical account of the growth of knowledge on caddisflies in Brazil. This example can be used to understand how growth of an area of study benefits from cooperation and investment.

## Materials and methods

To update the checklist we consulted the “Plataforma Lattes, base de dados de currículos do Conselho Nacional de Desenvolvimento Científico e Tecnológico (CNPq), Ministério da Ciência, Tecnologia e Inovação – Brazil", available in http://lattes.cnpq.br/, which is a federal database of scientists in Brazil. The researchers searched for the keywords Trichoptera, caddisflies, and caddisfly. Searches were concluded in September, 2014.

The search for compiling the Brazilian Trichoptera Checklist was performed on the following databases. The “*Web of Science*” was searched for Trichoptera and Brazil. All results from this search were examined. The other databases searched were “*The Trichoptera World Checklist”*, mantained by Dr. John C. Morse, Clemson University, USA; “*Brazilian Caddisflies: Checklist and Bibliography*”, mantained by Dr. Jorge L. Nessimian research team, Universidade Federal do Rio de Janeiro, Brazil; and “*Index to Organism Names*”, mantained by Thomson Reuters.

The acronyms used for Brazilian states are as listed: AC (Acre), AL (Alagoas), AM (Amazonas), AP (Amapá), BA (Bahia), CE (Ceará), DF (Distrito Federal), ES (Espírito Santo), GO (Goiás), MA (Maranhão), MG (Minas Gerais), MT (Mato Grosso), MS (Mato Grosso do Sul), PA (Pará), PB (Paraíba), PE (Pernambuco), PI (Piauí), PR (Paraná), RJ (Rio de Janeiro), RN (Rio Grande do Norte), RO (Rondônia), RR (Roraima), RS (Rio Grande do Sul), SC (Santa Catarina), SE (Sergipe), SP (São Paulo), TO (Tocantins). Brazil is divided in political regions as following: North (AC, AM, AP, PA, RO, RR, TO), Northeast (AL, BA, CE, MA, PB, PE, PI, RN, SE), Southeast (ES, MG, RJ, SP), Midwest (DF, GO, MS, MT) and South (PR, SC, RS).

State names presented in bold letters indicate a new state record from literature.

## Checklists

### Anomalopsychidae

#### Contulma

Flint, 1969

##### Notes

Flint Jr 1969a, Holzenthal and Flint Jr 1995

#### Contulma fluminensis

Holzenthal & Robertson, 2006

##### Distribution

**Rio de Janeiro**

##### Notes

Holzenthal and Robertson 2006

#### Contulma meloi

Holzenthal & Robertson, 2006

##### Distribution

**Sao Paulo**

##### Notes

Holzenthal and Robertson 2006

#### Contulma sana

Jardim & Nessimian, 2011

##### Distribution

**Rio de Janeiro**

##### Notes

Jardim and Nessimian 2011

#### Contulma tijuca

Holzenthal & Flint, 1995

##### Distribution

Rio de Janeiro

##### Notes

Holzenthal and Flint Jr 1995

#### Contulma tripui

Holzenthal & Robertson, 2006

##### Distribution

**Minas Gerais**

##### Notes

Holzenthal and Robertson 2006

### Atriplectididae

#### Neoatriplectides

Holzenthal, 1997

##### Notes

Holzenthal 1997

#### Neoatriplectides desiderata

Dumas & Nessimian, 2008

##### Distribution

**Minas Gerais**, **Rio de Janeiro**, **Sao Paulo**

##### Notes

Dumas and Nessimian 2008, Dumas and Nessimian 2012

### Calamoceratidae

#### Phylloicus

Müller, 1880

##### Notes

Müller 1880, Prather 2003

#### Phylloicus abdominalis

(Ulmer), 1905

##### Distribution

**Bahia**, **Ceara**, Minas Gerais, Parana, Rio de Janeiro, Santa Catarina, Sao Paulo

##### Notes

Ulmer 1905a, Prather 2003, Huamantico et al. 2005, Quinteiro et al. 2014

#### Phylloicus amazonas

Prather, 2003

##### Distribution

Amazonas

##### Notes

Prather 2003

#### Phylloicus angustior

Ulmer, 1905

##### Distribution

Goias, Minas Gerais, Parana, Rio Grande do Sul, Santa Catarina, **Sao Paulo**

##### Notes

Ulmer 1905b, Prather 2003, Calor 2011

#### Phylloicus auratus

Prather, 2003

##### Distribution

Amazonas

##### Notes

Prather 2003

#### Phylloicus bertioga

Prather, 2003

##### Distribution

Sao Paulo

##### Notes

Prather 2003

#### Phylloicus bidigitatus

Prather, 2003

##### Distribution

**Bahia**, **Ceara**, **Minas Gerais**, Rio de Janeiro, **Sao Paulo**

##### Notes

Prather 2003, Calor 2011, Quinteiro et al. 2014

#### Phylloicus bromeliarum

Müller, 1880

##### Distribution

Santa Catarina, Sao Paulo

##### Notes

Müller 1880, Prather 2003

#### Phylloicus brevior

Banks, 1915

##### Distribution

Parana, Rondonia

##### Notes

Banks 1915, Prather 2003

#### Phylloicus camargoi

Quinteiro & Calor, 2011

##### Distribution

**Sao Paulo**

##### Notes

Quinteiro et al. 2011

#### Phylloicus dumasi

Santos & Nessimian, 2010

##### Distribution

**Amazonas**

##### Notes

Santos and Nessimian 2010b

#### Phylloicus elektoros

Prather, 2003

##### Distribution

**Amazonas**

##### Notes

Prather 2003

#### Phylloicus fenestratus

Flint, 1974

##### Distribution

Amazonas, Paraiba, Parana, Rondonia, **Roraima**

##### Notes

Flint Jr 1974a, Prather 2003

#### Phylloicus flinti

Prather, 2003

##### Distribution

Rondonia

##### Notes

Prather 2003

#### Phylloicus major

Müller, 1880

##### Distribution

Rio de Janeiro, Santa Catarina, Sao Paulo

##### Notes

Müller 1880, Prather 2003

#### Phylloicus monneorum

Dumas & Nessimian, 2010

##### Distribution

**Bahia**, **Rio de Janeiro**

##### Notes

Dumas and Nessimian 2010b, Quinteiro et al. 2014

#### Phylloicus obliquus

Navás, 1931

##### Distribution

**Bahia**, **Ceara**, Minas Gerais, Rio de Janeiro, Santa Catarina

##### Notes

Navás 1931, Prather 2003, Quinteiro et al. 2014

#### Phylloicus paprockii

Prather, 2003

##### Distribution

**Bahia**, Minas Gerais

##### Notes

Prather 2003

#### Phylloicus plaumanni

Flint, 1983

##### Distribution

Santa Catarina

##### Notes

Flint Jr 1983a, Prather 2003

#### Phylloicus quadridigitatus

Prather, 2003

##### Distribution

Sao Paulo

##### Notes

Prather 2003

#### Phylloicus yolandae

Prather, 2003

##### Distribution

Parana

##### Notes

Prather 2003

### Ecnomidae

#### Austrotinodes

Schmid, 1955

##### Notes

Schmid 1955, Flint Jr 1973, Flint Jr and Denning 1989

#### Austrotinodes abrachium

Thomson & Holzenthal, 2010

##### Distribution

**Minas Gerais**

##### Notes

Thomson and Holzenthal 2010

#### Austrotinodes amazonensis

Flint & Denning, 1989

##### Distribution

Amazonas

##### Notes

Flint Jr and Denning 1989

#### Austrotinodes ariasi

Flint & Denning, 1989

##### Distribution

Amazonas, **Para**

##### Notes

Flint Jr and Denning 1989, Dumas et al. 2010

#### Austrotinodes belchioris

Thomson & Holzenthal, 2010

##### Distribution

**Minas Gerais**

##### Notes

Thomson and Holzenthal 2010

#### Austrotinodes bracteatus

Flint & Denning, 1989

##### Distribution

Sao Paulo

##### Notes

Flint Jr and Denning 1989

#### Austrotinodes longispinum

Thomson & Holzenthal, 2010

##### Distribution

**Rio de Janeiro**, **Sao Paulo**

##### Notes

Thomson and Holzenthal 2010

#### Austrotinodes paraguayensis

Flint, 1983

##### Distribution

Minas Gerais

##### Notes

Flint Jr 1983a

#### Austrotinodes prolixus

Flint & Denning, 1989

##### Distribution

Minas Gerais, **Rio de Janeiro**, **Sao Paulo**

##### Notes

Flint Jr and Denning 1989, Dumas et al. 2009, Calor 2011

#### Austrotinodes taquaralis

Thomson & Holzenthal, 2010

##### Distribution

**Rio de Janeiro**, **Minas Gerais**

##### Notes

Thomson and Holzenthal 2010

#### Austrotinodes uruguayensis

Angrisano, 1994

##### Distribution

Parana

##### Notes

Angrisano 1994, Blahnik et al. 2004

### Glossosomatidae

#### Canoptila

Mosely, 1939

##### Notes

Mosely 1939

#### Canoptila bifida

Mosely, 1939

##### Distribution

Santa Catarina

##### Notes

Mosely 1939

#### Canoptila williami

Robertson & Holzenthal, 2006

##### Distribution

**Sao Paulo**, **Parana**

##### Notes

Robertson and Holzenthal 2006

#### Itauara

Müller, 1888

##### Notes

Müller 1888, Flint Jr et al. 1999a, Robertson and Holzenthal 2011

#### Itauara alexanderi

Robertson & Holzenthal, 2011

##### Distribution

**Rio de Janeiro**

##### Notes

Robertson and Holzenthal 2011

#### Itauara amazonica

(Flint), 1971

##### Distribution

Amazonas

##### Notes

Flint Jr 1971, Robertson and Holzenthal 2011

#### Itauara blahniki

Robertson & Holzenthal, 2011

##### Distribution

**Sao Paulo**

##### Notes

Robertson and Holzenthal 2011

#### Itauara brasiliana

(Mosely), 1939

##### Distribution

**Santa Catarina**

##### Notes

Mosely 1939, Robertson and Holzenthal 2011

#### Itauara charlotta

Robertson & Holzenthal, 2011

##### Distribution

**Minas Gerais**

##### Notes

Robertson and Holzenthal 2011

#### Itauara emilia

Robertson & Holzenthal, 2011

##### Distribution

**Sao Paulo**

##### Notes

Robertson and Holzenthal 2011

#### Itauara flinti

Robertson & Holzenthal, 2011

##### Distribution

**Sao Paulo**

##### Notes

Robertson and Holzenthal 2011

#### Itauara jamesii

Robertson & Holzenthal, 2011

##### Distribution

**Minas Gerais**

##### Notes

Robertson and Holzenthal 2011

#### Itauara julia

Robertson & Holzenthal, 2011

##### Distribution

**Rio de Janeiro**

##### Notes

Robertson and Holzenthal 2011

#### Itauara lucinda

Robertson & Holzenthal, 2011

##### Distribution

**Minas Gerais**

##### Notes

Robertson and Holzenthal 2011

#### Itauara plaumanni

(Flint), 1974

##### Distribution

Santa Catarina

##### Notes

Flint Jr 1974b, Robertson and Holzenthal 2011

#### Itauara rodmani

Robertson & Holzenthal, 2011

##### Distribution

**Minas Gerais**

##### Notes

Robertson and Holzenthal 2011

#### Itauara simplex

Robertson & Holzenthal, 2011

##### Distribution

**Sao Paulo**

##### Notes

Robertson and Holzenthal 2011

#### Itauara stella

Robertson & Holzenthal, 2011

##### Distribution

**Sao Paulo**

##### Notes

Robertson and Holzenthal 2011

#### Itauara tusci

Robertson & Holzenthal, 2011

##### Distribution

**Rio de Janeiro**

##### Notes

Robertson and Holzenthal 2011

#### Mortoniella

Ulmer, 1906

##### Notes

Ulmer 1906, Mosely 1937, Flint Jr et al. 1999a, Flint Jr et al. 1999b, Blahnik and Holzenthal 2008

#### Mortoniella acauda

Blahnik & Holzenthal, 2011

##### Distribution

**Santa Catarina**

##### Notes

Blahnik and Holzenthal 2011

#### Mortoniella agosta

Blahnik & Holzenthal, 2011

##### Distribution

**Minas Gerais**, **Rio de Janeiro**

##### Notes

Blahnik and Holzenthal 2011

#### Mortoniella albolineata

Ulmer, 1907

##### Distribution

**Santa Catarina**, **Sao Paulo**

##### Notes

Ulmer 1907a, Blahnik and Holzenthal 2011

#### Mortoniella alicula

Blahnik & Holzenthal, 2011

##### Distribution

**Rio de Janeiro**

##### Notes

Blahnik and Holzenthal 2011

#### Mortoniella asymmetris

Blahnik & Holzenthal, 2011

##### Distribution

**Pernambuco**

##### Notes

Blahnik and Holzenthal 2011, Souza et al. 2013a

#### Mortoniella bocaina

Blahnik & Holzenthal, 2011

##### Distribution

**Sao Paulo**

##### Notes

Blahnik and Holzenthal 2011

#### Mortoniella catarinensis

(Flint), 1974

##### Distribution

**Santa Catarina**

##### Notes

Flint Jr 1974b, Blahnik and Holzenthal 2011

#### Mortoniella crescentis

Blahnik & Holzenthal, 2011

##### Distribution

**Rio de Janeiro**

##### Notes

Blahnik and Holzenthal 2011

#### Mortoniella dolonis

Blahnik & Holzenthal, 2011

##### Distribution

**Minas Gerais**, **Sao Paulo**

##### Notes

Blahnik and Holzenthal 2011

#### Mortoniella froehlichi

Blahnik & Holzenthal, 2011

##### Distribution

**Rio de Janeiro**

##### Notes

Blahnik and Holzenthal 2011

#### Mortoniella guahybae

Blahnik & Holzenthal, 2011

##### Distribution

**Sao Paulo**

##### Notes

Blahnik and Holzenthal 2011

#### Mortoniella hystricosa

Blahnik & Holzenthal, 2011

##### Distribution

**Santa Catarina**

##### Notes

Blahnik and Holzenthal 2011

#### Mortoniella intervales

Blahnik & Holzenthal, 2011

##### Distribution

**Sao Paulo**

##### Notes

Blahnik and Holzenthal 2011

#### Mortoniella latispina

Blahnik & Holzenthal, 2011

##### Distribution

**Rio de Janeiro**

##### Notes

Blahnik and Holzenthal 2011

#### Mortoniella longispina

Blahnik & Holzenthal, 2011

##### Distribution

**Santa Catarina**

##### Notes

Blahnik and Holzenthal 2011

#### Mortoniella meloi

Blahnik & Holzenthal, 2011

##### Distribution

**Sao Paulo**

##### Notes

Blahnik and Holzenthal 2011

#### Mortoniella ormina

(Mosely), 1939

##### Distribution

**Santa Catarina**

##### Notes

Mosely 1939, Blahnik and Holzenthal 2011

#### Mortoniella parauna

Blahnik & Holzenthal, 2011

##### Distribution

**Minas Gerais**

##### Notes

Blahnik and Holzenthal 2011

#### Mortoniella paraunota

Blahnik & Holzenthal, 2011

##### Distribution

**Santa Catarina**

##### Notes

Blahnik and Holzenthal 2011

#### Mortoniella pumila

Blahnik & Holzenthal, 2011

##### Distribution

**Minas Gerais**, **Rio de Janeiro**

##### Notes

Blahnik and Holzenthal 2011

#### Mortoniella pusilla

Blahnik & Holzenthal, 2011

##### Distribution

**Minas Gerais**

##### Notes

Blahnik and Holzenthal 2011

#### Mortoniella teutona

(Mosely), 1939

##### Distribution

**Minas Gerais**, **Parana**, **Rio de Janeiro**, **Santa Catarina**, **Sao Paulo**

##### Notes

Mosely 1939, Blahnik and Holzenthal 2011, Dumas and Nessimian 2012

#### Mortoniella tripuiensis

Blahnik & Holzenthal, 2011

##### Distribution

**Minas Gerais**

##### Notes

Blahnik and Holzenthal 2011

#### Mortoniella truncata

Blahnik & Holzenthal, 2011

##### Distribution

**Minas Gerais**

##### Notes

Blahnik and Holzenthal 2011

#### Mortoniella unota

(Mosely), 1939

##### Distribution

**Santa Catarina**

##### Notes

Mosely 1939, Blahnik and Holzenthal 2011

#### Mortoniella uruguaiensis

Blahnik & Holzenthal, 2011

##### Distribution

**Santa Catarina**

##### Notes

Blahnik and Holzenthal 2011

#### Protoptila

Banks, 1904

##### Notes

Banks 1904, Flint Jr 1971, Flint Jr 1996

#### Protoptila condylifera

Flint, 1971

##### Distribution

Amazonas

##### Notes

Flint Jr 1971

#### Protoptila cora

Flint, 1983

##### Distribution

Minas Gerais

##### Notes

Flint Jr 1983a, Blahnik et al. 2004

#### Protoptila disticha

Flint, 1971

##### Distribution

Amazonas, Para

##### Notes

Flint Jr 1971

#### Protoptila dubitans

Mosely, 1939

##### Distribution

Santa Catarina

##### Notes

Mosely 1939

#### Protoptila ensifera

Flint, 1971

##### Distribution

Amazonas, **Para**, **Roraima**

##### Notes

Flint Jr 1971, Flint Jr 1991

#### Protoptila flexispina

Flint, 1971

##### Distribution

Amazonas

##### Notes

Flint Jr 1971

#### Protoptila longispinata

Santos & Nessimian, 2009

##### Distribution

**Amazonas**, **Para**

##### Notes

Santos and Nessimian 2009c

#### Protoptila macilenta

Flint, 1971

##### Distribution

Para

##### Notes

Flint Jr 1971

#### Protoptila mara

Flint, 1971

##### Distribution

Amazonas

##### Notes

Flint Jr 1971

#### Protoptila simplex

Flint, 1971

##### Distribution

Amazonas, Para

##### Notes

Flint Jr 1971

#### Protoptila ternatia

Flint, 1971

##### Distribution

Amazonas

##### Notes

Flint Jr 1971

#### Protoptila tetravittata

Flint, 1971

##### Distribution

Amazonas

##### Notes

Flint Jr 1971

#### Protoptila trispicata

Flint, 1971

##### Distribution

Amazonas

##### Notes

Flint Jr 1971

#### Tolhuaca

Schmid, 1964

##### Notes

Schmid 1964

#### Tolhuaca brasiliensis

Robertson & Holzenthal, 2005

##### Distribution

**Sao Paulo**

##### Notes

Robertson and Holzenthal 2005

### Helicopsychidae

#### Helicopsyche

Siebold, 1856

##### Notes

Siebold 1856, Mülller 1885

#### Helicopsyche (Cochliopsyche) amica

Johanson, 2003

##### Distribution

**Para**

##### Notes

Johanson 2003

#### Helicopsyche (Cochliopsyche) amazona

Johanson, 2003

##### Distribution

**Amazonas**

##### Notes

Johanson 2003

#### Helicopsyche (Cochliopsyche) blahniki

Johanson, 2003

##### Distribution

**Amazonas**

##### Notes

Johanson 2003

#### Helicopsyche (Cochliopsyche) brazilia

Johanson, 2003

##### Distribution

**Minas Gerais**

##### Notes

Johanson 2003

#### Helicopsyche (Cochliopsyche) clara

(Ulmer), 1905

##### Distribution

**Amazonas**, Minas Gerais, **Pernambuco**, Santa Catarina, **Sao Paulo**

##### Notes

Ulmer 1905a, Flint Jr 1996, Johanson 2003, Blahnik et al. 2004, Souza et al. 2013a

#### Helicopsyche (Cochliopsyche) lobata

Flint, 1983

##### Distribution

Minas Gerais, Santa Catarina

##### Notes

Flint Jr 1983a, Blahnik et al. 2004

#### Helicopsyche (Cochliopsyche) opalescens

Flint, 1972

##### Distribution

**Amazonas**, **Minas Gerais**, **Mato Grosso**, **Para**, **Parana**, Rio de Janeiro, **Rondonia**, Roraima, **Santa Catarina**, Sao Paulo

##### Notes

Flint Jr 1972, Flint Jr 1996, Johanson 2003, Blahnik et al. 2004

#### Helicopsyche (Cochliopsyche) pandeirosa

Johanson, 2003

##### Distribution

**Minas Gerais**

##### Notes

Johanson 2003

#### Helicopsyche (Cochliopsyche) xinguensis

Johanson, 2003

##### Distribution

**Amazonas**, **Para**

##### Notes

Johanson 2003

#### Helicopsyche (Feropsyche) braziliensis

(Swainson), 1840

##### Distribution

Brazil

##### Notes

Swainson 1840

#### Helicopsyche (Feropsyche) cipoensis

Johanson & Malm, 2006

##### Distribution

**Minas Gerais**

##### Notes

Johanson and Malm 2006

#### Helicopsyche (Feropsyche) flinti

Johanson, 1999

##### Distribution

Santa Catarina

##### Notes

Johanson 1999

#### Helicopsyche (Feropsyche) helicoidella

(Vallot), 1855

##### Distribution

Bahia

##### Notes

Vallot 1855

#### Helicopsyche (Feropsyche) monda

Flint, 1983

##### Distribution

Minas Gerais, Parana, **Rio de Janeiro**, Santa Catarina, Sao Paulo

##### Notes

Flint Jr 1983a, Blahnik et al. 2004, Dumas et al. 2009

#### Helicopsyche (Feropsyche) muelleri

Banks, 1920

##### Distribution

Santa Catarina

##### Notes

Banks 1920

#### Helicopsyche (Feropsyche) paprockii

Johanson & Malm, 2006

##### Distribution

**Minas Gerais**

##### Notes

Johanson and Malm 2006

#### Helicopsyche (Feropsyche) planorboides

Machado, 1957

##### Distribution

Minas Gerais

##### Notes

Machado 1957

#### Helicopsyche (Feropsyche) valligera

Flint, 1983

##### Distribution

Santa Catarina

##### Notes

Flint Jr 1983a

#### Helicopsyche (Feropsyche) vergelana

Ross, 1956

##### Distribution

**Pernambuco**

##### Notes

Ross 1956a, Souza et al. 2013a

#### Helicopsyche (Feropsyche) timbira

Silva, Santos & Nessimian, 2014

##### Distribution

**Rio de Janeiro**, **Sao Paulo**

##### Notes

Silva et al. 2014

### Hydrobiosidae

#### Atopsyche

Banks, 1905

##### Notes

Banks 1905, Schmid 1989

#### Atopsyche (Atopsaura) acahuana

Schmid, 1989

##### Distribution

Espirito Santo, **Minas Gerais**, Rio de Janeiro

##### Notes

Schmid 1989, Blahnik et al. 2004

#### Atopsyche (Atopsaura) antisuya

Schmid, 1989

##### Distribution

Minas Gerais

##### Notes

Schmid 1989

#### Atopsyche (Atopsaura) apurimac

Schmid, 1989

##### Distribution

Rio de Janeiro

##### Notes

Schmid 1989

#### Atopsyche (Atopsaura) blahniki

Santos & Holzenthal, 2012

##### Distribution

**Rio de Janeiro**

##### Notes

Santos and Holzenthal 2012

#### Atopsyche (Atopsaura) galharada

Santos & Holzenthal, 2012

##### Distribution

**Sao Paulo**

##### Notes

Santos and Holzenthal 2012

#### Atopsyche (Atopsaura) hamata

Ross & King, 1952

##### Distribution

Roraima

##### Notes

Ross and King 1952

#### Atopsyche (Atopsaura) hatunpuna

Schmid, 1989

##### Distribution

**Minas Gerais**, Sao Paulo

##### Notes

Schmid 1989, Dumas and Nessimian 2012

#### Atopsyche (Atopsaura) huacachaca

Schmid, 1989

##### Distribution

Rio de Janeiro

##### Notes

Schmid 1989

#### Atopsyche (Atopsaura) huamachucu

Schmid, 1989

##### Distribution

Rio de Janeiro

##### Notes

Schmid 1989

#### Atopsyche (Atopsaura) huanapu

Schmid, 1989

##### Distribution

Rio de Janeiro, Sao Paulo

##### Notes

Schmid 1989, Blahnik et al. 2004

#### Atopsyche (Atopsaura) huarcu

Schmid, 1989

##### Distribution

Minas Gerais, Rio de Janeiro, Sao Paulo

##### Notes

Schmid 1989, Blahnik et al. 2004

#### Atopsyche (Atopsaura) longipennis

(Ulmer), 1905

##### Distribution

Santa Catarina

##### Notes

Ulmer 1905a

#### Atopsyche (Atopsaura) plancki

Marlier, 1964

##### Distribution

**Minas Gerais**, Rio de Janeiro, **Santa Catarina**, Sao Paulo

##### Notes

Marlier 1964b, Blahnik et al. 2004, Santos and Holzenthal 2012

#### Atopsyche (Atopsaura) sanctipauli

Flint, 1983

##### Distribution

**Minas Gerais**, Parana, Rio de Janeiro, Santa Catarina, Sao Paulo

##### Notes

Flint Jr 1983a, Blahnik et al. 2004

#### Atopsyche (Atopsaura) serica

Ross, 1953

##### Distribution

Santa Catarina

##### Notes

Ross 1953

#### Atopsyche (Atopsaura) siolii

Flint, 1971

##### Distribution

Amazonas

##### Notes

Flint Jr 1971

#### Atopsyche (Atopsaura) usingeri

Denning & Sykora, 1968

##### Distribution

Rio de Janeiro, **Sao Paulo**

##### Notes

Denning and Sykora 1968, Calor 2011

#### Atopsyche (Atopsaura) zernyi

Flint, 1974

##### Distribution

**Espirito Santo**, Minas Gerais, Rio deJaneiro, Santa Catarina, Sao Paulo

##### Notes

Flint Jr 1974b, Blahnik et al. 2004, Dumas et al. 2010

#### Atopsyche (Atopsyche) chirihuana

Schmid, 1989

##### Distribution

Minas Gerais

##### Notes

Schmid 1989, Blahnik et al. 2004

#### Atopsyche (Atopsyche) erigia

Ross, 1947

##### Distribution

Minas Gerais, **Sao Paulo**

##### Notes

Ross 1947, Blahnik et al. 2004, Calor 2011

#### Atopsyche (Atopsyche) parauna

Santos & Holzenthal, 2012

##### Distribution

**Minas Gerais**

##### Notes

Santos and Holzenthal 2012

#### Atopsyche (Atopsyche) urumarca

Schmid, 1989

##### Distribution

Minas Gerais, **Sao Paulo**

##### Notes

Schmid 1989, Santos and Holzenthal 2012

### Hydropsychidae

#### Blepharopus

Kolenati, 1859

##### Notes

Kolenati 1859

#### Blepharopus diaphanus

Kolenati, 1859

##### Distribution

**Espirito Santo**, Minas Gerais, **Mato Grosso**, Rio de Janeiro, **Pernambuco**, Parana, **Roraima**, Santa Catarina, **Sao Paulo**

##### Notes

Kolenati 1859, Flint Jr 1991, Marinoni and Almeida 2000, Blahnik et al. 2004, Dumas et al. 2009, Nogueira and Cabette 2011, Barcelos-Silva et al. 2012, Souza et al. 2013a, Costa et al. 2014

#### Centromacronema

Ulmer, 1905

##### Notes

Ulmer 1905a

#### Centromacronema auripenne

(Rambur), 1842

##### Distribution

**Minas Gerais**, **Rio de Janeiro**, **Santa Catarina**, **Sao Paulo**

##### Notes

Rambur 1842, Dumas et al. 2009, Dumas and Nessimian 2012

#### Centromacronema obscurum

(Ulmer), 1905

##### Distribution

Minas Gerais, Santa Catarina, Sao Paulo

##### Notes

Ulmer 1905a, Blahnik et al. 2004

#### Leptonema

Guérin-Méneville, 1843

##### Notes

Guérin-Méneville 1843, Flint et al. 1987

#### Leptonema agraphum

(Kolenati), 1859

##### Distribution

Rio de Janeiro

##### Notes

Kolenati 1859

#### Leptonema amazonense

Flint, 1978

##### Distribution

**Amazonas**, **Mato Grosso**, Roraima

##### Notes

Flint Jr 1978, Ribeiro et al. 2009, Nogueira and Cabette 2011

#### Leptonema amplifurcatum

Dumas & Nessimian, 2009

##### Distribution

**Rio de Janeiro**

##### Notes

Dumas and Nessimian 2009b

#### Leptonema aspersum

(Ulmer), 1907

##### Distribution

Bahia, Mato Grosso do Sul, **Mato Grosso**

##### Notes

Ulmer 1907c, Nogueira and Cabette 2011

#### Leptonema aterrimum

Mosely, 1933

##### Distribution

Para

##### Notes

Mosely 1933

#### Leptonema bifurcatodes

Flint, 2008

##### Distribution

**Rio de Janeiro**

##### Notes

Flint Jr 2008

#### Leptonema bifurcatum

Flint, McAlpine & Ross, 1987

##### Distribution

Espirito Santo, Minas Gerais

##### Notes

Flint et al. 1987, Blahnik et al. 2004

#### Leptonema boraceia

Flint, McAlpine & Ross, 1987

##### Distribution

Rio de Janeiro, Sao Paulo

##### Notes

Flint et al. 1987

#### Leptonema columbianum

Ulmer, 1905

##### Distribution

Amazonas, Bahia, Distrito Federal, Goias, Minas Gerais, Mato Grosso do Sul, Para, Rondonia, Sao Paulo

##### Notes

Ulmer 1905a

#### Leptonema crassum

Ulmer, 1905

##### Distribution

Espirito Santo, Goias, Minas Gerais, Mato Grosso, Roraima, Sao Paulo

##### Notes

Ulmer 1905a

#### Leptonema eugnathum

(Müller), 1921

##### Distribution

Santa Catarina

##### Notes

Müller 1921

#### Leptonema ferelunatum

Jardim, Dumas & Nessimian, 2010

##### Distribution

**Rio de Janeiro**

##### Notes

Jardim et al. 2010

#### Leptonema lacuniferum

Flint, 1978

##### Distribution

Amazonas

##### Notes

Flint Jr 1978

#### Leptonema lineaticorne

Flint, 2008

##### Distribution

**Minas Gerais**

##### Notes

Flint Jr 2008

#### Leptonema lunatum

Flint, McAlpine & Ross, 1987

##### Distribution

Santa Catarina

##### Notes

Flint et al. 1987

#### Leptonema macacu

Flint, 2008

##### Distribution

**Rio de Janeiro**

##### Notes

Flint Jr 2008

#### Leptonema maculatum

Mosely, 1933

##### Distribution

Amazonas, **Mato Grosso**, Para

##### Notes

Mosely 1933, Nogueira and Cabette 2011

#### Leptonema pallidum

Guérin-Meneville, 1843

##### Distribution

**Bahia**, **Ceara**, Distrito Federal, Espirito Santo, Goias, Minas Gerais, Rio de Janeiro, Sao Paulo

##### Notes

Guérin-Méneville 1843, Costa et al. 2014

#### Leptonema rostratum

Flint, McAlpine & Ross, 1987

##### Distribution

Amazonas, Mato Grosso, Para, Roraima

##### Notes

Schmid 1964, Flint et al. 1987, Costa et al. 2014

#### Leptonema sancticaroli

Flint, McAlpine & Ross, 1987

##### Distribution

**Amazonas**

##### Notes

Flint et al. 1987, Dumas et al. 2010

#### Leptonema santosi

Jardim, Dumas & Nessimian, 2010

##### Distribution

**Rio de Janeiro**

##### Notes

Jardim et al. 2010

#### Leptonema serranum

Navás, 1933

##### Distribution

Sao Paulo

##### Notes

Flint et al. 1987

#### Leptonema serratum

Jardim, Dumas & Nessimian, 2010

##### Distribution

**Rio de Janeiro**

##### Notes

Jardim et al. 2010

#### Leptonema sparsum

(Ulmer), 1905

##### Distribution

Amazonas, **Bahia**, Distrito Federal, Goias, Minas Gerais, Mato Grosso, Para, Parana, Rio de Janeiro, Rondonia, **Roraima**, Santa Catarina, Sao Paulo

##### Notes

Flint et al. 1987, Flint Jr 1991, Marinoni and Almeida 2000, Blahnik et al. 2004, Costa et al. 2014

#### Leptonema speciosum

(Burmeister), 1839

##### Distribution

Rio de Janeiro

##### Notes

Burmeister 1839

#### Leptonema spinulum

Flint, McAlpine & Ross, 1987

##### Distribution

**Amazonas**, Distrito Federal, Mato Grosso, **Para**

##### Notes

Flint et al. 1987, Dumas et al. 2010

#### Leptonema stigmaticum

Navás, 1916

##### Distribution

Rio de Janeiro

##### Notes

Burmeister 1839

#### Leptonema tholloni

Navás, 1923

##### Distribution

Rio de Janeiro

##### Notes

Flint et al. 1987

#### Leptonema tridens

Mosely, 1933

##### Distribution

Minas Gerais, Parana, Rio de Janeiro, Sao Paulo

##### Notes

Mosely 1933, Blahnik et al. 2004,

#### Leptonema trispicatum

Flint, McAlpine & Ross, 1987

##### Distribution

Parana, Sao Paulo

##### Notes

Flint et al. 1987, Blahnik et al. 2004

#### Leptonema viridianum

Navás, 1916

##### Distribution

**Bahia**, **Ceara**, **Espirito Santo**, Goias, Distrito Federal, Minas Gerais, **Para**, **Paraiba**, **Pernambuco**, **Parana**, Rio de Janeiro, **Sao Paulo**

##### Notes

Mosely 1933, Blahnik et al. 2004, Dumas and Nessimian 2012, Barcelos-Silva et al. 2012, Souza et al. 2013a, Costa et al. 2014

#### Macronema

Pictet, 1836

##### Notes

Pictet 1836, Flint Jr and Bueno-Soria 1982

#### Macronema amazonense

Flint, 1978

##### Distribution

Amazonas

##### Notes

Flint Jr 1978

#### Macronema argentilineatum

Ulmer, 1905

##### Distribution

Amazonas, Para

##### Notes

Ulmer 1905a

#### Macronema bicolor

Ulmer, 1905

##### Distribution

**Espirito Santo**, Minas Gerais, Santa Catarina, **Sao Paulo**

##### Notes

Ulmer 1905a, Dumas et al. 2010, Barcelos-Silva et al. 2012

#### Macronema burmeisteri

Banks, 1924

##### Distribution

Amazonas

##### Notes

Banks 1924

#### Macronema exophtalmum

Flint, 1978

##### Distribution

Amazonas

##### Notes

Flint Jr 1978

#### Macronema fragile

Banks, 1915

##### Distribution

Amazonas

##### Notes

Banks 1915

#### Macronema fulvum

Ulmer, 1905

##### Distribution

Rio de Janeiro, **Sao Paulo**

##### Notes

Ulmer 1905a, Calor 2011

#### Macronema hageni

Banks, 1924

##### Distribution

Amazonas, Minas Gerais, **Mato Grosso**, Para, **Roraima**

##### Notes

Banks 1924, Flint Jr 1991, Blahnik et al. 2004, Nogueira and Cabette 2011

#### Macronema immaculatum

Mosely, 1934

##### Distribution

Parana, Sao Paulo

##### Notes

Mosely 1934a

#### Macronema lachlani

Banks, 1924

##### Distribution

Amazonas

##### Notes

Banks 1924

#### Macronema lineatum

Pictet, 1836

##### Distribution

Bahia

##### Notes

Pictet 1836

#### Macronema muelleri

Banks, 1924

##### Distribution

Amazonas

##### Notes

Banks 1924

#### Macronema partitum

Navás, 1932

##### Distribution

Rio de Janeiro

##### Notes

Navás 1932b

#### Macronema parvum

Ulmer, 1905

##### Distribution

Amazonas

##### Notes

Ulmer 1905a

#### Macronema pennyi

Flint, 1978

##### Distribution

Amazonas

##### Notes

Flint Jr 1978

#### Macronema percitans

Walker, 1860

##### Distribution

Amazonas, Mato Grosso, Para

##### Notes

Walker 1860

#### Macronema pertyi

Banks, 1924

##### Distribution

Mato Grosso, Para

##### Notes

Banks 1924

#### Macronema rubiginosum

Guérin-Meneville, 1843

##### Distribution

Brazil

##### Notes

Guérin-Méneville 1843

#### Macrostemum

Kolenati, 1859

##### Notes

Kolenati 1859, Flint Jr and Bueno-Soria 1982, França et al. 2013

#### Macrostemum arcuatum

(Erichson), 1848

##### Distribution

Amazonas, **Mato Grosso**, Para

##### Notes

Erichson 1848, Nogueira and Cabette 2011, França et al. 2013

#### Macrostemum brasiliense

(Fischer, 1970)

##### Distribution

Bahia, **Espirito Santo**, **Minas Gerais**, **Rio de Janeiro**, Sao Paulo

##### Notes

Perty 1833, Fischer 1970, Dumas and Nessimian 2012, França et al. 2013

#### Macrostemum braueri

(Banks), 1924

##### Distribution

Amazonas

##### Notes

Banks 1924, França et al. 2013

#### Macrostemum bravoi

França, Paprocki & Calor, 2013

##### Distribution

**Bahia**, **Mato Grosso**

##### Notes

França et al. 2013

#### Macrostemum digramma

(McLachlan), 1871

##### Distribution

**Espirito Santo**, Minas Gerais, **Rio de Janeiro**, Sao Paulo

##### Notes

McLachlan 1871, Calor 2011, Barcelos-Silva et al. 2012, França et al. 2013

#### Macrostemum erichsoni

(Banks), 1920

##### Distribution

**Acre**, Amazonas, **Bahia**, **Espirito Santo**, **Para**

##### Notes

Banks 1920, Barcelos-Silva et al. 2012, França et al. 2013

#### Macrostemum hyalinum

(Pictet), 1836

##### Distribution

**Acre**, **Bahia**, **Ceara**, **Espirito Santo**, **Mato Grosso**, Para, **Paraiba**, **Pernambuco**, Parana, **Rio de Janeiro**, **Sao Paulo**

##### Notes

Pictet 1836, Marinoni and Almeida 2000, Dumas et al. 2009, Calor 2011, Nogueira and Cabette 2011, França et al. 2013

#### Macrostemum negrense

(Flint), 1978

##### Distribution

Amazonas, **Mato Grosso**, Para

##### Notes

Flint Jr 1978, França et al. 2013

#### Macrostemum nigrum

França, Paprocki & Calor, 2013

##### Distribution

**Bahia**

##### Notes

França et al. 2013

#### Macrostemum par

(Navás), 1930

##### Distribution

Sao Paulo

##### Notes

Navás 1930, França et al. 2013

#### Macrostemum santaeritae

(Ulmer), 1905

##### Distribution

**Acre**, Amazonas, **Bahia**, **Mato Grosso**, Para

##### Notes

Ulmer 1905b, Nogueira and Cabette 2011, França et al. 2013

#### Macrostemum surinamense

(Flint), 1974

##### Distribution

Amazonas

##### Notes

Flint Jr 1974a, França et al. 2013

#### Macrostemum ulmeri

(Banks), 1913

##### Distribution

**Acre**, Amazonas, Minas Gerais, **Mato Grosso**, Para, **Pernambuco**, **Rondonia**, Roraima, **Sao Paulo**

##### Notes

Banks 1913, Blahnik et al. 2004, Nogueira and Cabette 2011, França et al. 2013, Souza et al. 2013a

#### Plectromacronema

Ulmer, 1906

##### Notes

Ulmer 1906, Flint Jr 1967, Flint Jr 1983b

#### Plectromacronema comptum

Ulmer, 1906

##### Distribution

Amazonas, Para, **Roraima**

##### Notes

Ulmer 1906, Flint Jr 1991

#### Plectromacronema subfuscum

(Banks), 1920

##### Distribution

Santa Catarina

##### Notes

Banks 1920

#### Pseudomacronema

Ulmer, 1905

##### Notes

Ulmer 1905a

#### Pseudomacronema vittatum

Ulmer, 1905

##### Distribution

Amazonas, Para

##### Notes

Ulmer 1905a

#### Smicridea

McLachlan, 1871

##### Notes

McLachlan 1871, Flint Jr 1974a, Flint Jr 1989

#### Smicridea (Rhyacophylax) abrupta

Flint, 1974

##### Distribution

Amazonas, **Mato Grosso**, **Roraima**

##### Notes

Flint Jr 1974a, Flint Jr 1991, Nogueira and Cabette 2011

#### Smicridea (Rhyacophylax) appendiculata

Flint, 1972

##### Distribution

Amazonas, Minas Gerais, **Mato Grosso**, **Roraima**

##### Notes

Flint Jr 1972, Blahnik et al. 2004, Albino et al. 2011

#### Smicridea (Rhyacophylax) araguaiense

Albino, Pes & Hamada, 2011

##### Distribution

**MatoGrosso**

##### Notes

Albino et al. 2011

#### Smicridea (Rhyacophylax) atrobasis

Flint, 1983

##### Distribution

Santa Catarina

##### Notes

Flint Jr 1983a

#### Smicridea (Rhyacophylax) bicornuta

Albino, Pes & Hamada, 2011

##### Distribution

**Mato Grosso**

##### Notes

Albino et al. 2011

#### Smicridea (Rhyacophylax) bifasciata

Albino, Pes & Hamada, 2011

##### Distribution

**Mato Grosso**

##### Notes

Albino et al. 2011

#### Smicridea (Rhyacophylax) brasiliana

(Ulmer), 1905

##### Distribution

Santa Catarina

##### Notes

Ulmer 1905a

#### Smicridea (Rhyacophylax) caligata

Flint, 1974

##### Distribution

Amazonas

##### Notes

Flint Jr 1974a

#### Smicridea (Rhyacophylax) columbiana

(Ulmer), 1905

##### Distribution

Amazonas, **Roraima**

##### Notes

Ulmer 1905a, Flint Jr 1991

#### Smicridea (Rhyacophylax) coronata

Flint, 1980

##### Distribution

**Espirito Santo**, Minas Gerais, **Mato Grosso**, Sao Paulo

##### Notes

Flint Jr 1980, Albino et al. 2011, Barcelos-Silva et al. 2012

#### Smicridea (Rhyacophylax) dentifera

Flint, 1983

##### Distribution

Sao Paulo

##### Notes

Flint Jr 1983a, Blahnik et al. 2004

#### Smicridea (Rhyacophylax) dentisserrata

Albino, Pes & Hamada, 2011

##### Distribution

**Mato Grosso**

##### Notes

Albino et al. 2011

#### Smicridea (Rhyacophylax) discalis

Flint, 1972

##### Distribution

Minas Gerais, Parana

##### Notes

Flint Jr 1972, Marinoni and Almeida 2000, Blahnik et al. 2004

#### Smicridea (Rhyacophylax) ephippifer

Flint, 1978

##### Distribution

**Mato Grosso**, Para

##### Notes

Flint Jr 1978, Nogueira and Cabette 2011

#### Smicridea (Rhyacophylax) flinti

Albino, Pes & Hamada, 2011

##### Distribution

**Amazonas**, **Mato Grosso**

##### Notes

Albino et al. 2011

#### Smicridea (Rhyacophylax) forcipata

Flint, 1983

##### Distribution

Santa Catarina

##### Notes

Flint Jr 1983a, Blahnik et al. 2004

#### Smicridea (Rhyacophylax) froehlichi

Almeida & Flint, 2002

##### Distribution

**Minas Gerais**, Rio de Janeiro, **Sao Paulo**

##### Notes

Almeida and Flint Jr 2002, Dumas et al. 2010

#### Smicridea (Rhyacophylax) gladiator

Flint, 1978

##### Distribution

Amazonas

##### Notes

Flint Jr 1978

#### Smicridea (Rhyacophylax) helenae

Albino, Pes & Hamada, 2011

##### Distribution

**Amazonas**, **Mato Grosso**, **Roraima**

##### Notes

Albino et al. 2011

#### Smicridea (Rhyacophylax) iguazu

Flint, 1983

##### Distribution

**Espirito Santo**, Minas Gerais, Parana, Rio de Janeiro, Santa Catarina

##### Notes

Flint Jr 1983a, Marinoni and Almeida 2000, Barcelos-Silva et al. 2012

#### Smicridea (Rhyacophylax) jundiai

Almeida & Flint, 2002

##### Distribution

Espirito Santo, **Minas Gerais**, Parana, Rio de Janeiro, **Sao Paulo**

##### Notes

Almeida and Flint Jr 2002, Dumas et al. 2010

#### Smicridea (Rhyacophylax) mangaratiba

Almeida & Flint, 2002

##### Distribution

Rio de Janeiro

##### Notes

Almeida and Flint Jr 2002

#### Smicridea (Rhyacophylax) marlieri

Flint, 1978

##### Distribution

Amazonas, Para

##### Notes

Flint Jr 1978

#### Smicridea (Rhyacophylax) marua

Flint, 1978

##### Distribution

Amazonas

##### Notes

Flint Jr 1978

#### Smicridea (Rhyacophylax) mesembrina

Navás, 1918

##### Distribution

**Mato Grosso**

##### Notes

Navás 1918, Nogueira and Cabette 2011

#### Smicridea (Ryachophylax) palmar

Sganga, 2005

##### Distribution

**Espirito Santo**, **Pernambuco**

##### Notes

Barcelos-Silva et al. 2012, Souza et al. 2013a

#### Smicridea (Rhyacophylax) piraya

Flint, 1983

##### Distribution

**Espirito Santo**, Minas Gerais, Parana, Santa Catarina, Sao Paulo

##### Notes

Flint Jr 1983a, Marinoni and Almeida 2000, Blahnik et al. 2004, Barcelos-Silva et al. 2012

#### Smicridea (Rhyacophylax) pseudolobata

Flint, 1978

##### Distribution

Amazonas, **Para**

##### Notes

Flint Jr 1978, Dumas et al. 2010

#### Smicridea (Rhyacophylax) radula

Flint, 1974

##### Distribution

**Espirito Santo**, Minas Gerais, Parana, Rio de Janeiro, **Santa Catarina**, Sao Paulo

##### Notes

Flint Jr 1974c, Blahnik et al. 2004, Dumas et al. 2009, Barcelos-Silva et al. 2012

#### Smicridea (Rhyacophylax) ralphi

Almeida & Flint, 2002

##### Distribution

Espirito Santo, Parana, Rio de Janeiro, Sao Paulo

##### Notes

Almeida and Flint Jr 2002

#### Smicridea (Rhyacophylax) roraimense

Albino, Pes & Hamada, 2011

##### Distribution

**Espirito Santo**, **Pernambuco**, **Roraima**

##### Notes

Albino et al. 2011, Barcelos-Silva et al. 2012, Souza et al. 2013a

#### Smicridea (Rhyacophylax) scutellaris

Flint, 1974

##### Distribution

Amazonas, Minas Gerais, Para, **Roraima**

##### Notes

Flint Jr 1974a, Flint Jr 1991, Blahnik et al. 2004

#### Smicridea (Rhyacophylax) spinulosa

Flint, 1972

##### Distribution

Amazonas, Para, Parana, Santa Catarina, Sao Paulo

##### Notes

Flint Jr 1972, Marinoni and Almeida 2000, Blahnik et al. 2004

#### Smicridea (Rhyacophylax) unguiculata

Flint, 1983

##### Distribution

Goias, Minas Gerais, Parana, Santa Catarina, Sao Paulo

##### Notes

Flint Jr 1983a, Marinoni and Almeida 2000, Blahnik et al. 2004

#### Smicridea (Rhyacophylax) vermiculata

Flint, 1978

##### Distribution

Minas Gerais, Para, Parana, Santa Catarina, **Sao Paulo**

##### Notes

Flint Jr 1978, Marinoni and Almeida 2000, Blahnik et al. 2004, Calor 2011

#### Smicridea (Rhyacophylax) vilela

Flint, 1978

##### Distribution

Amazonas, Para

##### Notes

Flint Jr 1978

#### Smicridea (Rhyacophylax) voluta

Flint, 1978

##### Distribution

Amazonas, Para

##### Notes

Flint Jr 1978

#### Smicridea (Rhyacophylax) weidneri

Flint, 1972

##### Distribution

Parana, Santa Catarina

##### Notes

Flint Jr 1972, Marinoni and Almeida 2000

#### Smicridea (Smicridea) aequalis

Banks, 1920

##### Distribution

Para

##### Notes

Banks 1920

#### Smicridea (Smicridea) albosignata

Ulmer, 1907

##### Distribution

Minas Gerais, Parana, Rio de Janeiro, Sao Paulo

##### Notes

Ulmer 1907a, Marinoni and Almeida 2000, Blahnik et al. 2004

#### Smicridea (Smicridea) bivittata

(Hagen), 1861

##### Distribution

Minas Gerais, Para, **Sao Paulo**

##### Notes

Hagen 1861, Blahnik et al. 2004, Calor 2011

#### Smicridea (Smicridea) gemina

Blahnik, 1995

##### Distribution

**Espirito Santo**

##### Notes

Blahnik 2005, Barcelos-Silva et al. 2012

#### Smicridea (Smicridea) mirnae

Almeida & Flint, 2002

##### Distribution

Parana

##### Notes

Almeida and Flint Jr 2002

#### Smicridea (Smicridea) obliqua

Flint, 1974

##### Distribution

Amazonas

##### Notes

Flint Jr 1974a

#### Smicridea (Smicridea) palifera

Flint, 1981

##### Distribution

**Espirito Santo**, **Mato Grosso**, **Pernambuco**, Rio de Janeiro, **Roraima**

##### Notes

Flint Jr 1981, Blahnik et al. 2004, Albino et al. 2011, Nogueira and Cabette 2011, Barcelos-Silva et al. 2012, Souza et al. 2013a

#### Smicridea (Smicridea) paranensis

Flint, 1983

##### Distribution

Minas Gerais, Parana, Rio de Janeiro, **Sao Paulo**

##### Notes

Flint Jr 1983a, Marinoni and Almeida 2000, Blahnik et al. 2004, Calor 2011

#### Smicridea (Smicridea) reinerti

Flint, 1978

##### Distribution

Para

##### Notes

Flint Jr 1978

#### Smicridea (Smicridea) sattleri

Denning & Sykora, 1968

##### Distribution

Sao Paulo

##### Notes

Denning and Sykora 1968

#### Smicridea (Smicridea) sexspinosa

Flint, 1978

##### Distribution

Amazonas

##### Notes

Flint Jr 1978

#### Smicridea (Smicridea) truncata

Flint, 1974

##### Distribution

Amazonas, **Mato Grosso**, Para, **Rio de Janeiro**

##### Notes

Flint Jr 1974a, Dumas et al. 2009, Nogueira and Cabette 2011

#### Synoestropsis

Ulmer, 1905

##### Notes

Ulmer 1905a, Flint Jr et al. 1999b

#### Synoestropsis furcata

Flint, 1974

##### Distribution

**Mato Grosso**, Para

##### Notes

Flint Jr 1974a, Flint Jr 1991, Calor 2008a

#### Synoestropsis grisoli

Navás, 1924

##### Distribution

Amazonas, **Bahia**, Minas Gerais, **Mato Grosso**, Para, **Roraima**

##### Notes

Navás 1924, Flint Jr 1991, Blahnik et al. 2004, Nogueira and Cabette 2011, Costa et al. 2014

#### Synoestropsis obliqua

Ulmer, 1905

##### Distribution

Rio Grande do Sul

##### Notes

Ulmer 1905a

#### Synoestropsis pedicillata

Ulmer, 1905

##### Distribution

**Espirito Santo**, Minas Gerais, **Pernambuco**, Santa Catarina, **Sao Paulo**

##### Notes

Ulmer 1905a, Blahnik et al. 2004, Calor 2011, Barcelos-Silva et al. 2012, Souza et al. 2013a

#### Synoestropsis punctipennis

Ulmer, 1905

##### Distribution

Amazonas

##### Notes

Ulmer 1905a

#### Synoestropsis stictonota

Navás, 1932

##### Distribution

Santa Catarina

##### Notes

Navás 1932a

### Hydroptilidae

#### Abtrichia

Mosely, 1939

##### Notes

Mosely 1939

#### Abtrichia antennata

Mosely, 1939

##### Distribution

Minas Gerais, **Pernambuco**, **Rio de Janeiro**, Santa Catarina, Minas Gerais

##### Notes

Mosely 1939, Blahnik et al. 2004, Dumas et al. 2010

#### Abtrichia squamosa

Mosely, 1939

##### Distribution

Minas Gerais, Rio de Janeiro, Santa Catarina

##### Notes

Mosely 1939, Blahnik et al. 2004

#### Acostatrichia

Mosely, 1939

##### Notes

Mosely 1939

#### Acostatrichia brevipenis

Flint, 1974

##### Distribution

Roraima

##### Notes

Flint Jr 1974a

#### Acostatrichia plaumanni

Mosely, 1939

##### Distribution

Santa Catarina

##### Notes

Mosely 1939

#### Acostatrichia simulans

Mosely, 1939

##### Distribution

Santa Catarina

##### Notes

Mosely 1939

#### Alisotrichia

Flint, 1964

##### Distribution

Rondonia

##### Notes

Flint Jr 1964

#### Alisotrichia cacaulandia

Harris & Flint, 2002

##### Distribution

Rondonia

##### Notes

Harris and Flint Jr 2002

#### Alisotrichia holzenthali

Santos, 2011

##### Distribution

**Minas Gerais**

##### Notes

Santos 2011

#### Alisotrichia macae

Santos, 2011

##### Distribution

**Rio de Janeiro**

##### Notes

Santos 2011

#### Alisotrichia nessimiani

Santos, 2011

##### Distribution

**Rio de Janeiro**

##### Notes

Santos 2011

#### Alisotrichia ubatuba

Santos, 2011

##### Distribution

**Sao Paulo**

##### Notes

Santos 2011

#### Anchitrichia

Flint, 1970

##### Notes

Flint Jr 1970

#### Anchitrichia duplifurcata

Flint, 1983

##### Distribution

Minas Gerais, Rio de Janeiro

##### Notes

Flint Jr 1983a, Guahyba 1991, Blahnik et al. 2004

#### Ascotrichia

Flint, 1983

##### Notes

Flint Jr 1983a

#### Ascotrichia frontalis

Flint, 1983

##### Distribution

Espirito Santo, Rio de Janeiro

##### Notes

Flint Jr 1983a

#### Betrichia

Mosely, 1939

##### Notes

Mosely 1939

#### Betrichia alibrachia

Thomson, 2012

##### Distribution

**Rio de Janeiro**

##### Notes

Thomson 2012

#### Betrichia hamulifera

Flint, 1983

##### Distribution

**Pernambuco**, **Sao Paulo**, **Santa Catarina**

##### Notes

Flint Jr 1983a, Calor 2011, Souza et al. 2013b

#### Betrichia longistyla

Flint, 1983

##### Distribution

Santa Catarina

##### Notes

Flint Jr 1983a

#### Betrichia zilbra

Mosely, 1939

##### Distribution

Santa Catarina

##### Notes

Mosely 1939

#### Bredinia

Flint, 1968

##### Notes

Flint Jr 1968

#### Bredinia espinosa

Harris, Holzenthal & Flint, 2002

##### Distribution

Mato Grosso, Rondonia

##### Notes

Harris et al. 2002b

#### Byrsopteryx

Flint, 1981

##### Notes

Flint Jr 1981

#### Byrsopteryx abrelata

Harris & Holzenthal, 1994

##### Distribution

Parana, Rio de Janeiro, **Sao Paulo**

##### Notes

Harris and Holzenthal 1994, Blahnik et al. 2004, Santos and Nessimian 2010a

#### Byrsopteryx carioca

Santos & Nessimian, 2010

##### Distribution

**Riode Janeiro**

##### Notes

Santos and Nessimian 2010a

#### Byrsopteryx espinhosa

Harris & Holzenthal, 1994

##### Distribution

Rio de Janeiro

##### Notes

Harris and Holzenthal 1994

#### Ceratotrichia

Flint, 1992

#### Ceratotrichia sp.

##### Distribution

**Amazonas**, **Rio Grande do Sul**

##### Notes

Flint Jr 1992, Pes and Hamada 2004, Spies et al. 2006

#### Costatrichia

Mosely, 1937

##### Notes

Mosely 1937

#### Costatrichia fluminensis

Santos & Nessimian, 2010

##### Distribution

**Rio de Janeiro**

##### Notes

Santos and Nessimian 2010c

#### Costatrichia nelsonferreirai

Santos & Nessimian, 2010

##### Distribution

**Para**

##### Notes

Santos and Nessimian 2010c

#### Costatrichia noite

Angrisano, 1995

##### Distribution

**Amazonas**

##### Notes

Angrisano 1994, Santos et al. 2013

#### Costatrichia ipixuna

Santos, Takyia & Nessimian, 2013

##### Distribution

**Amazonas**

##### Notes

Santos et al. 2013

#### Dicaminus

Müller, 1879

##### Notes

Müller 1879b

#### Dicaminus ladislavii

Müller, 1879

##### Distribution

Santa Catarina

##### Notes

Müller 1879b

#### Eutonella

Müller, 1921

##### Notes

Müller 1921, Flint Jr et al. 1999b

#### Eutonella peltopsychodes

Müller, 1921

##### Distribution

Santa Catarina

##### Notes

Müller 1921

#### Flintiella

Angrisano, 1995

##### Notes

Angrisano 1995

#### Flintiella andreae

Angrisano, 1995

##### Distribution

**Pernambuco**

##### Notes

Angrisano 1995, Souza et al. 2013b

#### Flintiella astilla

Harris, Flint & Holzenthal, 2002

##### Distribution

**Bahia**, **Mato Grosso**, **Parana**, **Sao Paulo**

##### Notes

Harris et al. 2002a

#### Flintiella boraceia

Harris, Flint & Holzenthal, 2002

##### Distribution

Sao Paulo

##### Notes

Harris et al. 2002a

#### Flintiella carajas

Santos, Jardim & Nessimian, 2011

##### Distribution

**Para**

##### Notes

Santos et al. 2011

#### Flintiella manauara

Santos & Nessimian, 2009

##### Distribution

**Amazonas**

##### Notes

Santos and Nessimian 2009a

#### Flintiella pizotensis

Harris, Flint & Holzenthal, 2002

##### Distribution

**Amazonas**

##### Notes

Dumas et al. 2010

#### Hydroptila

Dalman, 1819

##### Notes

Dalman 1819, Bueno-Soria 1984, Harris and Holzenthal 1999

#### Hydroptila argentinica

Flint, 1983

##### Distribution

Parana, **Rio de Janeiro**, Sao Paulo

##### Notes

Flint Jr 1983a, Blahnik et al. 2004, Dumas et al. 2009

#### Hydroptila producta

Mosely, 1939

##### Distribution

**Rio de Janeiro**, Santa Catarina

##### Notes

Mosely 1939, Dumas et al. 2009

#### Leucotrichia

Mosely, 1934

##### Notes

Mosely 1934b, Flint Jr 1970

#### Leucotrichia bicornuta

Thomson, 2012

##### Distribution

**Rio de Janeiro**

##### Notes

Thomson 2012

#### Leucotrichia brasiliana

Sattler & Sykora, 1977

##### Distribution

Amazonas

##### Notes

Sattler and Sykora 1977

#### Metrichia

Ross, 1938

##### Notes

Ross 1938

#### Metrichia pernambucana

Souza & Santos, 2013

##### Distribution

**Pernambuco**

##### Notes

Souza et al. 2013b

#### Metrichia sp.

##### Distribution

**Amazonas**, **Goias**, **Rio de Janeiro**, **Rio Grande do Sul**, **Sao Paulo**

##### Notes

Pes et al. 2005, Spies et al. 2006, Martins-Silva et al. 2008, Spies and Froehlich 2009, Dumas and Nessimian 2012

#### Neotrichia

Morton, 1905

##### Notes

Morton 1905

#### Neotrichia abbreviata

Flint, 1983

##### Distribution

Santa Catarina

##### Notes

Flint Jr 1983a

#### Neotrichia bellinii

Santos & Nessimian, 2009

##### Distribution

**Amazonas**

##### Notes

Santos and Nessimian 2009b

#### Neotrichia browni

Harris, 1990

##### Distribution

**Amazonas**

##### Notes

Harris 1990, Santos and Nessimian 2009b

#### Neotrichia colmillosa

Harris, 1990

##### Distribution

**Amazonas**

##### Notes

Harris 1990, Santos and Nessimian 2009b

#### Neotrichia didii

Santos & Nessimian, 2009

##### Distribution

**Amazonas**

##### Notes

Santos and Nessimian 2009b

#### Neotrichia djalmasantosi

Santos & Nessimian, 2009

##### Distribution

**Amazonas**

##### Notes

Santos and Nessimian 2009b

#### Neotrichia dubitans

(Mosely), 1939

##### Distribution

**Rio de Janeiro**, Santa Catarina

##### Notes

Mosely 1939, Dumas et al. 2009

#### Neotrichia durior

Flint, 1983

##### Distribution

Santa Catarina

##### Notes

Flint Jr 1983a

#### Neotrichia feolai

Santos & Nessimian, 2009

##### Distribution

**Amazonas**, **Pernambuco**

##### Notes

Santos and Nessimian 2009b, Souza et al. 2013b

#### Neotrichia filifera

Flint, 1983

##### Distribution

Minas Gerais, **Pernambuco**

##### Notes

Flint Jr 1983a, Blahnik et al. 2004, Souza et al. 2013b

#### Neotrichia garrinchai

Santos & Nessimian, 2009

##### Distribution

**Amazonas**

##### Notes

Santos and Nessimian 2009b

#### Neotrichia gilmari

Santos & Nessimian, 2009

##### Distribution

**Amazonas**

##### Notes

Santos and Nessimian 2009b

#### Neotrichia longissima

Flint, 1983

##### Distribution

Santa Catarina

##### Notes

Flint Jr 1983a

#### Neotrichia niltonsantosi

Santos & Nessimian, 2009

##### Distribution

**Amazonas**

##### Notes

Santos and Nessimian 2009b

#### Neotrichia noteuna

(Mosely), 1939

##### Distribution

Santa Catarina

##### Notes

Mosely 1939

#### Neotrichia novara

(Mosely), 1939

##### Distribution

Santa Catarina

##### Notes

Mosely 1939

#### Neotrichia orlandoi

Santos & Nessimian, 2009

##### Distribution

**Amazonas**

##### Notes

Santos and Nessimian 2009b

#### Neotrichia ovona

(Mosely), 1939

##### Distribution

Santa Catarina

##### Notes

Mosely 1939

#### Neotrichia pelei

Santos & Nessimian, 2009

##### Distribution

**Amazonas**

##### Notes

Santos and Nessimian 2009b

#### Neotrichia rotundata

Flint, 1974

##### Distribution

Roraima

##### Notes

Flint Jr 1974a

#### Neotrichia sicilicula

Flint, 1983

##### Distribution

Santa Catarina

##### Notes

Flint Jr 1983a

#### Neotrichia tertia

(Mosely), 1939

##### Distribution

Santa Catarina

##### Notes

Mosely 1939

#### Neotrichia teutonia

Flint, 1983

##### Distribution

Santa Catarina

##### Notes

Flint Jr 1983a

#### Neotrichia vavai

Santos & Nessimian, 2009

##### Distribution

**Amazonas**

##### Notes

Santos and Nessimian 2009b

#### Neotrichia zagalloi

Santos & Nessimian, 2009

##### Distribution

**Amazonas**

##### Notes

Santos and Nessimian 2009b

#### Neotrichia zitoi

Santos & Nessimian, 2009

##### Distribution

**Amazonas**

##### Notes

Santos and Nessimian 2009b

#### Nothotrichia

Flint, 1967

##### Notes

Flint Jr 1967, Harris and Armitage 1997

#### Nothotrichia tupi

Holzenthal & Harris, 2002

##### Distribution

Brazil

##### Notes

Holzenthal and Harris 2002

#### Ochrotrichia

Mosely, 1934

##### Notes

Mosely 1934b

#### Ochrotrichia caatinga

Souza, Santos & Takiya, 2014

##### Distribution

**Ceará**

##### Notes

Souza et al. 2014

#### Ochrotrichia concha

Bueno & Santiago, 1992

##### Distribution

Amazonas

##### Notes

Bueno-Soria and Santiago-Fragoso 1992

#### Ochrotrichia constricta

Souza, Santos & Takiya, 2014

##### Distribution

**Bahia**

##### Notes

Souza et al. 2014

#### Ochrotrichia igrapiuna

Souza, Santos & Takiya, 2014

##### Distribution

**Bahia**

##### Notes

Souza et al. 2014

#### Ochrotrichia limeirai

Souza, Santos & Takiya, 2014

##### Distribution

**Ceara**

##### Notes

Souza et al. 2014

#### Ochrotrichia manuensis

Flint & Bueno-Soria

##### Distribution

**Bahia**

##### Notes

Souza et al. 2014

#### Ochrotrichia patulosa

(Wasmund & Holzenthal), 2007

##### Distribution

**Ceara**, **Rio de Janeiro**

##### Notes

Dumas et al. 2009, Oláh and Johanson 2011, Quinteiro et al. 2014

#### Ochrotrichia priapo

Souza, Santos & Takiya, 2014

##### Distribution

**Bahia**

##### Notes

Souza et al. 2014

#### Oxyethira

Eaton, 1873

##### Notes

Eaton 1873

#### Oxyethira bettyae

Thomson & Holzenthal, 2012

##### Distribution

**Pernambuco**

##### Notes

Thomson and Holzenthal 2012, Souza et al. 2013b

#### Oxyethira bicornuta

Kelley, 1983

##### Distribution

Amazonas

##### Notes

Kelley 1983

#### Oxyethira brasiliensis

Kelley, 1983

##### Distribution

Para

##### Notes

Kelley 1983

#### Oxyethira circaverna

Kelley, 1983

##### Distribution

**Amazonas**

##### Notes

Kelley 1983, Santos et al. 2009

#### Oxyethira discaelata

Kelley, 1983

##### Distribution

Amazonas

##### Notes

Kelley 1983

#### Oxyethira espinada

Holzenthal & Harris, 1992

##### Distribution

Minas Gerais

##### Notes

Harris and Holzenthal 1992, Dumas et al. 2009, Blahnik et al. 2004

#### Oxyethira hyalina

Müller, 1879

##### Distribution

Santa Catarina

##### Notes

Müller 1879b

#### Oxyethira lagunita

Flint, 1983

##### Distribution

Parana

##### Notes

Flint Jr 1983a

#### Oxyethira longipenis

Santos, Henriques-Oliveira & Nessimian, 2009

##### Distribution

**Amazonas**

##### Notes

Santos et al. 2009

#### Oxyethira longissima

Flint, 1974

##### Distribution

**Amazonas**

##### Notes

Flint Jr 1974a, Santos et al. 2009

#### Oxyethira luanae

Santos, Henriques-Oliveira & Nessimian, 2009

##### Distribution

**Amazonas**

##### Notes

Santos et al. 2009

#### Oxyethira macrosterna

Flint, 1974

##### Distribution

**Amazonas**

##### Notes

Flint Jr 1974a, Santos et al. 2009

#### Oxyethira merga

Kelley, 1983

##### Distribution

Roraima

##### Notes

Kelley 1983

#### Oxyethira parce

(Edwards & Arnold), 1961

##### Distribution

Minas Gerais

##### Notes

Edwards and Arnold 1961, Blahnik et al. 2004

#### Oxyethira peruviana

Harris & Davenport, 1999

##### Distribution

**Amazonas**

##### Notes

Harris and Davenport 1999, Santos et al. 2009

#### Oxyethira picita

Harris & Davenport, 1999

##### Distribution

**Amazonas**

##### Notes

Harris and Davenport 1999, Santos et al. 2009

#### Oxyethira presilla

Harris & Davenport, 1999

##### Distribution

**Amazonas**

##### Notes

Harris and Davenport 1999, Santos et al. 2009

#### Oxyethira santiagensis

Flint, 1982

##### Distribution

Brazil

##### Notes

Flint Jr 1982b

#### Oxyethira sinistra

Santos, Henriques-Oliveira & Nessimian, 2009

##### Distribution

**Amazonas**

##### Notes

Santos et al. 2009

#### Oxyethira spirogyrae

Müller, 1879

##### Distribution

Santa Catarina

##### Notes

Müller 1879b

#### Oxyethira spissa

Kelley, 1983

##### Distribution

Amazonas, Para

##### Notes

Kelley 1983

#### Oxyethira tica

Holzenthal & Harris, 1992

##### Distribution

**Amazonas**, Minas Gerais, **Rio de Janeiro**

##### Notes

Harris and Holzenthal 1992, Blahnik et al. 2004, Santos et al. 2009, Dumas and Nessimian 2012

#### Oxyethira zilaba

(Mosely), 1939

##### Distribution

Minas Gerais, Parana, Santa Catarina, Sao Paulo

##### Notes

Mosely 1939, Blahnik et al. 2004

#### Peltopsyche

Müller, 1879

##### Notes

Müller 1879b, Flint Jr 1971

#### Peltopsyche maclachlani

Müller, 1879

##### Distribution

Santa Catarina

##### Notes

Müller 1879b

#### Peltopsyche sieboldii

Müller, 1879 sieboldi em Morse

##### Distribution

Santa Catarina

##### Notes

Müller 1879b

#### Rhyacopsyche

Müller, 1879

##### Notes

Müller 1879b

#### Rhyacopsyche angra

Santos, Jardim & Nessimian, 2011

##### Distribution

**Rio de Janeiro**

##### Notes

Santos et al. 2009

#### Rhyacopsyche bulbosa

Wasmund & Holzenthal, 2007

##### Distribution

**Minas Gerais**, **Rio de Janeiro**

##### Notes

Wasmund and Holzenthal 2007

#### Rhyacopsyche diacantha

Santos, Jardim & Nessimian, 2011

##### Distribution

**Para**

##### Notes

Santos et al. 2011

#### Rhyacopsyche dikrosa

Wasmund & Holzenthal, 2007

##### Distribution

**Minas Gerais**, **Rio de Janeiro**, **Sao Paulo**

##### Notes

Wasmund and Holzenthal 2007

#### Rhyacopsyche hagenii

Müller, 1879

##### Distribution

**Parana**, **Rio de Janeiro**, Santa Catarina, **Sao Paulo**

##### Notes

Müller 1879b, Wasmund and Holzenthal 2007, Dumas et al. 2009

#### Taraxitrichia

Flint & Harris, 1992

#### Taraxitrichia sp.

##### Distribution

**Amazonas**

##### Notes

Flint Jr and Harris 1992, Pes and Hamada 2003

#### Tricholeiochiton

Kloet & Hincks, 1944

##### Notes

Kloet and Hincks 1944

#### Tricholeiochiton neotropicalis

Flint, 1992

##### Distribution

Roraima

##### Notes

Flint Jr 1992

#### Zumatrichia

Mosely, 1937

##### Notes

Mosely 1937

#### Zumatrichia sp.

##### Distribution

**Amazonas**

##### Notes

Mosely 1937, Pes and Hamada 2004

### Leptoceridae

#### Achoropsyche

Holzenthal, 1984

##### Notes

Holzenthal 1984

#### Achoropsyche duodecimpunctata

(Navás), 1916

##### Distribution

Amazonas, Espirito Santo, Minas Gerais, **Mato Grosso**, Para, Parana, Rio de Janeiro, **Rondonia**, Roraima, Santa Catarina, Sao Paulo

##### Notes

Navás 1916a, Almeida and Marinoni 2000, Blahnik et al. 2004, Dumas et al. 2009, Nogueira and Cabette 2011, Quinteiro et al. 2014

#### Amazonatolica

Holzenthal & Pes, 2004

##### Notes

Holzenthal and Pes 2004

#### Amazonatolica hamadae

Holzenthal & Pes, 2004

##### Distribution

**Amazonas**, **Bahia**, **Mato Grosso**, **Rondonia**

##### Notes

Holzenthal and Pes 2004, Nogueira and Cabette 2011

#### Atanatolica

Mosely, 1936

##### Notes

Mosely 1936, Holzenthal 1988b

#### Atanatolica brasiliana

(Brauer), 1865

##### Distribution

Rio de Janeiro, **Sao Paulo**

##### Notes

Brauer 1865, Calor 2011

#### Atanatolica flinti

Holzenthal, 1988

##### Distribution

Rio de Janeiro

##### Notes

Holzenthal 1988b

#### Grumichella

Müller, 1879

##### Notes

Müller 1879b, Holzenthal 1988b

#### Grumichella aequiunguis

Flint, 1983

##### Distribution

Minas Gerais, Parana, Santa Catarina

##### Notes

Flint Jr 1983a, Blahnik et al. 2004

#### Grumichella rostrata

Thienemann, 1905

##### Distribution

Minas Gerais, Rio de Janeiro, Sao Paulo, Santa Catarina

##### Notes

Thienemann 1905, Holzenthal 1988b

#### Nectopsyche

Müller, 1879

##### Notes

Müller 1879b

#### Nectopsyche adusta

Flint, 1983

##### Distribution

**Sao Paulo**

##### Notes

Flint Jr 1983a, Calor 2011

#### Nectopsyche aureovittata

Flint, 1983

##### Distribution

Minas Gerais, Parana, Rio de Janeiro, Santa Catarina, Sao Paulo

##### Notes

Flint Jr 1983a, Almeida and Marinoni 2000, Blahnik et al. 2004

#### Nectopsyche acutiloba

Flint, 1974

##### Distribution

Minas Gerais

##### Notes

Flint Jr 1974a, Blahnik et al. 2004

#### Nectopsyche bella

(Müller), 1921

##### Distribution

Santa Catarina

##### Notes

Müller 1921

#### Nectopsyche bruchi

(Navás), 1920

##### Distribution

Minas Gerais, Parana, **Rio de Janeiro**

##### Notes

Navás 1920, Dumas et al. 2009

#### Nectopsyche brunneofascia

Flint, 1983

##### Distribution

Sao Paulo, Santa Catarina

##### Notes

Flint Jr 1983a, Blahnik et al. 2004

#### Nectopsyche diminuta

(Banks), 1920

##### Distribution

Amazonas, Para, Roraima

##### Notes

Banks 1920, Flint Jr 1991

#### Nectopsyche flavofasciata

(Ulmer), 1907

##### Distribution

**Minas Gerais**, Santa Catarina

##### Notes

Ulmer 1907a, Blahnik et al. 2004

#### Nectopsyche fuscomaculata

Flint, 1983

##### Distribution

**Minas Gerais**, **Pernambuco**, Parana, Rio de Janeiro, Santa Catarina, **Sao Paulo**

##### Notes

Flint Jr 1983a, Almeida and Marinoni 2000, Blahnik et al. 2004, Dumas and Nessimian 2012, Souza et al. 2013a

#### Nectopsyche gemma

(Müller), 1880

##### Distribution

Santa Catarina

##### Notes

Müller 1880

#### Nectopsyche gemmoides

Flint, 1981

##### Distribution

**Sao Paulo**

##### Notes

Flint Jr 1981, Calor 2011

#### Nectopsyche jenseni

(Ulmer), 1905

##### Distribution

Amazonas

##### Notes

Ulmer 1905b

#### Nectopsyche modesta

Müller, 1921

##### Distribution

Santa Catarina

##### Notes

Müller 1921

#### Nectopsyche muelleri

(Ulmer), 1905

##### Distribution

Santa Catarina

##### Notes

Ulmer 1905a

#### Nectopsyche muhni

(Navás), 1916

##### Distribution

Minas Gerais, **Rio de Janeiro**, **Roraima**, **Sao Paulo**

##### Notes

Navás 1916b, Flint Jr 1991, Blahnik et al. 2004, Dumas et al. 2009, Calor 2011

#### Nectopsyche multilineata

Flint, 1983

##### Distribution

**Roraima**

##### Notes

Flint Jr 1983a, Flint Jr 1991

#### Nectopsyche navasi

Holzenthal, 1999

##### Distribution

Santa Catarina

##### Notes

Flint Jr et al. 1999a

#### Nectopsyche nigricapilla

(Navás), 1922

##### Distribution

**MatoGrosso**

##### Notes

Nogueira and Cabette 2011

#### Nectopsyche ortizi

Holzenthal, 1995

##### Distribution

Minas Gerais, Para, Parana, Rio de Janeiro,

##### Notes

Holzenthal 1995, Almeida and Marinoni 2000, Blahnik et al. 2004

#### Nectopsyche pantosticta

Flint, 1983

##### Distribution

Rio de Janeiro, Rio Grande do Sul, **Sao Paulo**

##### Notes

Flint Jr 1983a, Blahnik et al. 2004, Calor 2011

#### Nectopsyche punctata

(Ulmer), 1905

##### Distribution

Minas Gerais, Para, **Rio de Janeiro**, **Roraima**, Sao Paulo

##### Notes

Ulmer 1905b, Flint Jr 1991, Blahnik et al. 2004, Dumas et al. 2009

#### Nectopsyche quatourguttata

(Navás), 1922

##### Distribution

**Mato Grosso**

##### Notes

Nogueira and Cabette 2011

#### Nectopsyche separata

(Banks), 1920

##### Distribution

Minas Gerais, Parana, Rio de Janeiro, Santa Catarina, Sao Paulo

##### Notes

Banks 1920, Almeida and Marinoni 2000, Blahnik et al. 2004

#### Nectopsyche splendida

(Navás), 1917

##### Distribution

**Bahia**, Minas Gerais, **Piaui**, Parana, **Roraima**

##### Notes

Navás 1917, Flint Jr 1991, Almeida and Marinoni 2000, Blahnik et al. 2004, Quinteiro et al. 2014

#### Neoathripsodes

Holzenthal, 1989

##### Notes

Holzenthal 1989

#### Neoathripsodes anomalus

Holzenthal, 1989

##### Distribution

Minas Gerais, Rio de Janeiro, **Sao Paulo**

##### Notes

Holzenthal 1984, Calor 2011

#### Notalina

Mosely, 1936

##### Notes

Mosely 1936, Holzenthal 1988b

#### Notalina (Neonotalina) brasiliana

Holzenthal, 1986

##### Distribution

Minas Gerais

##### Notes

Holzenthal 1986

#### Notalina (Neonotalina) cipo

Holzenthal, 1986

##### Distribution

Minas Gerais

##### Notes

Holzenthal 1986

#### Notalina (Neonotalina) froehlichi

Calor & Holzenthal, 2006

##### Distribution

**Minas Gerais**

##### Notes

Calor et al. 2006

#### Notalina (Neonotalina) goianensis

Calor, 2008

##### Distribution

**Goias**

##### Notes

Calor 2008b

#### Notalina (Neonotalina) hamiltoni

Holzenthal, 1986

##### Distribution

**Minas Gerais**, **Rio de Janeiro**, Sao Paulo

##### Notes

Holzenthal 1986, Dumas and Nessimian 2012

#### Notalina (Neonotalina) jordanensis

Henriques-Oliveira, Spies & Dumas, 2012

##### Distribution

**Minas Gerais**, **Sao Paulo**

##### Notes

Henriques-Oliveira et al. 2012

#### Notalina (Neonotalina) morsei

Holzenthal, 1986

##### Distribution

Minas Gerais, **Rio de Janeiro**, **Sao Paulo**

##### Notes

Holzenthal 1986, Calor et al. 2006, Calor 2011

#### Notalina (Neonotalina) paulista

Calor & Holzenthal, 2006

##### Distribution

Rio de Janeiro, Sao Paulo

##### Notes

Calor et al. 2006

#### Oecetis

McLachlan, 1877

##### Notes

McLachlan 1877

#### Oecetis amazonica

(Banks), 1924

##### Distribution

Amazonas

##### Notes

Banks 1924

#### Oecetis angelae

Henriques-Oliveria, Dumas & Nessimian, 2014

##### Distribution

**Mato Grosso do Sul**

##### Notes

Henriques-Oliveira et al. 2014

#### Oecetis connata

Flint, 1974

##### Distribution

**Amazonas**, **Para**

##### Notes

Flint Jr 1974a, Dumas et al. 2010, Quinteiro et al. 2014

#### Oecetis danielae

Henriques-Oliveria, Dumas & Nessimian, 2014

##### Distribution

**Amazonas**

##### Notes

Henriques-Oliveira et al. 2014

#### Oecetis excisa

Ulmer, 1907

##### Distribution

**Bahia**, **Ceara**, **Pernambuco**, **Sao Paulo**

##### Notes

Ulmer 1907a, Calor 2011, Souza et al. 2013a, Quinteiro et al. 2014

#### Oecetis fibra

Chen & Morse, 2012

##### Distribution

**Sao Paulo**

##### Notes

Quinteiro and Calor 2012

#### Oecetis iara

Henriques-Oliveria, Dumas & Nessimian, 2014

##### Distribution

**Parana**

##### Notes

Henriques-Oliveira et al. 2014

#### Oecetis inconspicua

(Walker), 1852

##### Distribution

**Bahia**, Minas Gerais, **Paraiba**, Parana, **Piaui**, **Sao Paulo**

##### Notes

Walker 1852, Blahnik et al. 2004, Calor 2011, Quinteiro et al. 2014

#### Oecetis iguazu

Flint, 1983

##### Distribution

**Bahia**, Espirito Santo, **Minas Gerais**, Rio de Janeiro, Santa Catarina, Sao Paulo

##### Notes

Flint Jr 1983a, Blahnik et al. 2004, Quinteiro et al. 2014

#### Oecetis paranensis

Flint, 1982

##### Distribution

**Bahia**, Minas Gerais, **Pernambuco**

##### Notes

Flint Jr 1982a, Souza et al. 2013a, Quinteiro et al. 2014

#### Oecetis punctipennis

(Ulmer), 1905

##### Distribution

Bahia, Minas Gerais, **Sao Paulo**, **Pernambuco**, **Roraima**

##### Notes

Ulmer 1905b, Flint Jr 1991, Calor 2011, Souza et al. 2013a

#### Oecetis rafaeli

Flint, 1991

##### Distribution

Roraima

##### Notes

Flint Jr 1991

#### Setodes

Rambur, 1842

##### Notes

Rambur 1842

#### Setodes sp.

##### Distribution

**Goias**

##### Notes

Barbosa et al. 2011

#### Triplectides

Kolenati, 1859

##### Notes

Kolenati 1859

#### Triplectides egleri

Sattler, 1963

##### Distribution

Amazonas, Para

##### Notes

Sattler 1963

#### Triplectides gracilis

(Burmeister), 1839

##### Distribution

**Bahia**, Espirito Santo, Minas Gerais, Rio de Janeiro, Parana, Santa Catarina, Sao Paulo

##### Notes

Burmeister 1839, Holzenthal 1988a, Almeida and Marinoni 2000, Quinteiro et al. 2014

#### Triplectides itatiaia

Dumas & Nessimian, 2010

##### Distribution

**Rio de Janeiro**

##### Notes

Dumas and Nessimian 2010a

#### Triplectides misionensis

Holzenthal, 1988

##### Distribution

**Minas Gerais**, Parana, Rio de Janeiro, Santa Catarina, Sao Paulo

##### Notes

Holzenthal 1988a, Blahnik et al. 2004, Dumas et al. 2010

#### Triplectides neotropicus

Holzenthal, 1988

##### Distribution

Minas Gerais, **Rio de Janeiro**, **Sao Paulo**

##### Notes

Holzenthal 1988a, Blahnik et al. 2004, Dumas et al. 2009, Calor 2011

#### Triplectides ultimus

Holzenthal, 1988

##### Distribution

**Minas Gerais**, Rio de Janeiro

##### Notes

Holzenthal 1988a, Dumas et al. 2010

### Limnephilidae

#### Antarctoecia

Ulmer, 1907

##### Notes

Ulmer 1907b

#### Antarctoecia brasiliensis

Huamantinco & Nessimian, 2003

##### Distribution

Minas Gerais

##### Notes

Huamantinco and Nessimian 2003, Huamantinco and Nessimian 2004a

### Odontoceridae

#### Anastomoneura

Huamantinco & Nessimian, 2004

#### Anastomoneura guahybae

Huamantinco & Nessimian, 2004

##### Distribution

**Minas Gerais**

##### Notes

Huamantinco and Nessimian 2004b

#### Barypenthus

Burmeister, 1839

##### Notes

Burmeister 1839, Flint Jr 1969a, Paprocki and Holzenthal 2002

#### Barypenthus concolor

Burmeister, 1839

##### Distribution

**Bahia**, Minas Gerais, Rio de Janeiro, Sao Paulo

##### Notes

Burmeister 1839, Paprocki and Holzenthal 2002, Quinteiro et al. 2014

#### Marilia

Müller, 1880

##### Notes

Müller 1880

#### Marilia aiuruoca

Dumas & Nessimian, 2009

##### Distribution

**Minas Gerais**, **Rio de Janeiro**

##### Notes

Dumas and Nessimian 2009a

#### Marilia alata

Flint, 1974

##### Distribution

**Amazonas**, **Pernambuco**

##### Notes

Flint Jr 1974a, Souza et al. 2013a

#### Marilia albicornis

(Burmeister), 1839

##### Distribution

**Sao Paulo**

##### Notes

Burmeister 1839, Calor 2011

#### Marilia elongata

Martynov, 1912

##### Distribution

Minas Gerais

##### Notes

Martynov 1912, Blahnik et al. 2004

#### Marilia fasciculata

Banks, 1913

##### Distribution

**Pernambuco**, Rondonia

##### Notes

Banks 1913, Souza et al. 2013a

#### Marilia flexuosa

Ulmer, 1905

##### Distribution

Brazil

##### Notes

Ulmer 1905b

#### Marilia guaira

Flint, 1983

##### Distribution

Goias, **Roraima**

##### Notes

Flint Jr 1983a, Flint Jr 1991

#### Marilia huamantincoae

Dumas & Nessimian, 2009

##### Distribution

**Rio de Janeiro**

##### Notes

Dumas and Nessimian 2009a

#### Marilia infundibulum

Flint, 1983

##### Distribution

Santa Catarina

##### Notes

Flint Jr 1983a

#### Marilia lateralis

Flint, 1983

##### Distribution

Mato Grosso do Sul

##### Notes

Flint Jr 1983a

#### Marilia major

Müller, 1880

##### Distribution

Minas Gerais, Parana, **Rio de Janeiro**, Santa Catarina

##### Notes

Müller 1880, Blahnik et al. 2004, Dumas and Nessimian 2009a

#### Marilia minor

Müller, 1880

##### Distribution

Minas Gerais, Rio de Janeiro, Santa Catarina

##### Notes

Müller 1880, Blahnik et al. 2004

#### Marilia sioli

Marlier, 1964

##### Distribution

Amazonas

##### Notes

Marlier 1964b

#### Marilia truncata

Flint, 1983

##### Distribution

Minas Gerais

##### Notes

Flint Jr 1983a, Blahnik et al. 2004

### Philopotamidae

#### Alterosa

Blahnik, 2005

##### Notes

Flint Jr 1971, Almeida and Duarte 2003, Blahnik 2005

#### Alterosa affinis

Dumas & Nessimian, 2013

##### Distribution

**Espirito Santo**, **Minas Gerais**

##### Notes

Dumas and Nessimian 2013

#### Alterosa amadoi

Dumas, Calor & Nessimian, 2013

##### Distribution

**Bahia**

##### Notes

Dumas et al. 2013

#### Alterosa bandeira

Dumas & Nessimian, 2013

##### Distribution

**Espirito Santo**, **Minas Gerais**

##### Notes

Dumas and Nessimian 2013

#### Alterosa beckeri

Blahnik, 2005

##### Distribution

**Minas Gerais**, **Rio de Janeiro**

##### Notes

Blahnik 2005, Dumas et al. 2010

#### Alterosa bilanceolata

Dumas & Nessimian, 2013

##### Distribution

**Parana**

##### Notes

Dumas and Nessimian 2013

#### Alterosa bocainae

Blahnik, 2005

##### Distribution

**Sao Paulo**

##### Notes

Blahnik 2005

#### Alterosa boraceiae

Blahnik, 2005

##### Distribution

**Sao Paulo**

##### Notes

Blahnik 2005

#### Alterosa caissara

Dumas & Nessimian, 2013

##### Distribution

**Sao Paulo**

##### Notes

Dumas and Nessimian 2013

#### Alterosa caparaonensis

Blahnik, 2005

##### Distribution

**Espirito Santo**, **Minas Gerais**

##### Notes

Blahnik 2005, Dumas and Nessimian 2013

#### Alterosa capixaba

Dumas & Nessimian, 2013

##### Distribution

**Espirito Santo**

##### Notes

Dumas and Nessimian 2013

#### Alterosa castroalvesi

Dumas, Calor & Nessimian, 2013

##### Distribution

**Bahia**

##### Notes

Dumas et al. 2013

#### Alterosa catarinae

Dumas & Nessimian, 2013

##### Distribution

**Santa Catarina**

##### Notes

Dumas and Nessimian 2013

#### Alterosa caymmii

Dumas, Calor & Nessimian, 2013

##### Distribution

**Bahia**

##### Notes

Dumas et al. 2013

#### Alterosa escova

Blahnik, 2005

##### Distribution

**Rio de Janeiro**, **Sao Paulo**

##### Notes

Blahnik 2005

#### Alterosa falcata

Blahnik, 2005

##### Distribution

**Minas Gerais**, **Riode Janeiro**, **Sao Paulo**

##### Notes

Blahnik 2005

#### Alterosa fimbriata

Blahnik, 2005

##### Distribution

**Rio de Janeiro**

##### Notes

Blahnik 2005

#### Alterosa flinti

Blahnik, 2005

##### Distribution

**Espirito Santo**, **Minas Gerais**, **Rio de Janeiro**

##### Notes

Blahnik 2005, Dumas and Nessimian 2013

#### Alterosa fluminensis

Blahnik, 2005

##### Distribution

**Rio de Janeiro**

##### Notes

Blahnik 2005

#### Alterosa graciosa

Dumas & Nessimian, 2013

##### Distribution

**Parana**

##### Notes

Dumas and Nessimian 2013

#### Alterosa guapimirim

Blahnik, 2005

##### Distribution

**Rio de Janeiro**

##### Notes

Blahnik 2005

#### Alterosa holzenthali

Blahnik, 2005

##### Distribution

**Santa Catarina**

##### Notes

Blahnik 2005

#### Alterosa inappendiculata

Dumas & Nessimian, 2013

##### Distribution

**Parana**

##### Notes

Dumas and Nessimian 2013

#### Alterosa intervales

Blahnik, 2005

##### Distribution

**Parana**, **Sao Paulo**

##### Notes

Blahnik 2005, Dumas and Nessimian 2013

#### Alterosa itatiaiae

Blahnik, 2005

##### Distribution

**Rio de Janeiro**

##### Notes

Blahnik 2005

#### Alterosa jordaensis

Blahnik, 2005

##### Distribution

**Sao Paulo**

##### Notes

Blahnik 2005

#### Alterosa marinonii

(Almeida & Duarte), 2003

##### Distribution

**Parana**, **Sao Paulo**

##### Notes

Almeida and Duarte 2003, Blahnik 2005, Dumas and Nessimian 2013

#### Alterosa morato

Dumas & Nessimian, 2013

##### Distribution

**Parana**

##### Notes

Dumas and Nessimian 2013

#### Alterosa nessimiani

Jardim & Dumas, 2012

##### Distribution

**Rio de Janeiro**

##### Notes

Jardim and Dumas 2012

#### Alterosa orgaosensis

Blahnik, 2005

##### Distribution

**Rio de Janeiro**

##### Notes

Blahnik 2005

#### Alterosa paprockii

Blahnik, 2005

##### Distribution

**Minas Gerais**

##### Notes

Blahnik 2005

#### Alterosa paranaensis

Dumas & Nessimian, 2013

##### Distribution

**Parana**

##### Notes

Dumas and Nessimian 2013

#### Alterosa ruschii

Dumas & Nessimian, 2013

##### Distribution

**Espirito Santo**

##### Notes

Dumas and Nessimian 2013

#### Alterosa sanctaeteresae

Blahnik, 2005

##### Distribution

**Espirito Santo**

##### Notes

Blahnik 2005

#### Alterosa sanctipauli

(Flint), 1971

##### Distribution

**Sao Paulo**

##### Notes

Flint Jr 1971, Blahnik 2005

#### Alterosa schadrackorum

Blahnik, 2005

##### Distribution

**Santa Catarina**

##### Notes

Blahnik 2005

#### Alterosa spiesae

Dumas & Nessimian, 2013

##### Distribution

**Sao Paulo**

##### Notes

Dumas and Nessimian 2013

#### Alterosa tripuiensis

Blahnik, 2005

##### Distribution

**Minas Gerais**

##### Notes

Blahnik 2005

#### Alterosa truncata

Blahnik, 2005

##### Distribution

**Espirito Santo**, **Minas Gerais**, **Rio de Janeiro**, **Sao Paulo**

##### Notes

Blahnik 2005, Dumas and Nessimian 2012, Barcelos-Silva et al. 2012

#### Chimarra

Stephens, 1829

##### Notes

Stephens 1829, Flint Jr 1998, Blahnik 1998

#### Chimarra (Chimarra) adamsae

Blahnik, 1998

##### Distribution

Minas Gerais, Para, Parana, Sao Paulo

##### Notes

Blahnik 1998, Blahnik et al. 2004

#### Chimarra (Chimarra) calori

Blahnik & Holzenthal, 2012

##### Distribution

**Minas Gerais**, **Sao Paulo**

##### Notes

Blahnik and Holzenthal 2012

#### Chimarra (Chimarra) uara

Flint, 1971

##### Distribution

Amazonas, Distrito Federal, Minas Gerais, **Para**, **Pernambuco**, Rondonia, Santa Catarina

##### Notes

Flint Jr 1971, Blahnik et al. 2004, Dumas et al. 2010, Souza et al. 2013a

#### Chimarra (Chimarrita) akantha

Blahnik, 1997

##### Distribution

Amazonas

##### Notes

Blahnik 1997

#### Chimarra (Chimarrita) camella

Blahnik, 1997

##### Distribution

Minas Gerais, Rio de Janeiro, Sao Paulo

##### Notes

Blahnik 1997, Blahnik et al. 2004

#### Chimarra (Chimarrita) camura

Blahnik, 1997

##### Distribution

Rio de Janeiro, Sao Paulo

##### Notes

Blahnik 1997, Blahnik et al. 2004

#### Chimarra (Chimarrita) chela

Blahnik, 1997

##### Distribution

**Amazonas**

##### Notes

Blahnik 1997, Santos and Nessimian 2009d

#### Chimarra (Chimarrita) curvipenis

Blahnik & Holzenthal, 2012

##### Distribution

**Minas Gerais**

##### Notes

Blahnik and Holzenthal 2012

#### Chimarra (Chimarrita) heligma

Blahnik, 1997

##### Distribution

Minas Gerais

##### Notes

Blahnik 1997

#### Chimarra (Chimarrita) kontilos

Blahnik, 1997

##### Distribution

Espirito Santo, Minas Gerais, Rio de Janeiro, Sao Paulo

##### Notes

Blahnik 1997, Blahnik et al. 2004

#### Chimarra (Chimarrita) latiforceps

Blahnik & Holzenthal, 2012

##### Distribution

**Minas Gerais**, **Sao Paulo**

##### Notes

Blahnik and Holzenthal 2008

#### Chimarra (Chimarrita) majuscula

Blahnik, 1997

##### Distribution

Rio de Janeiro, Sao Paulo

##### Notes

Blahnik 1997, Blahnik et al. 2004

#### Chimarra (Chimarrita) simpliciforma

Flint, 1971

##### Distribution

Amazonas

##### Notes

Flint Jr 1971

#### Chimarra (Chimarrita) tortuosa

Blahnik, 1997

##### Distribution

Amazonas

##### Notes

Blahnik 1997

#### Chimarra (Chimarrita) xingu

Blahnik, 1997

##### Distribution

Para

##### Notes

Blahnik 1997

#### Chimarra (Curgia) aurivittata

Flint, 1971

##### Distribution

Amazonas, Rondonia

##### Notes

Flint Jr 1971, Flint Jr 1998

#### Chimarra (Curgia) beckeri

Flint, 1998

##### Distribution

**Espirito Santo**, **Minas Gerais**, Rio de Janeiro, **Sao Paulo**

##### Notes

Flint Jr 1998, Dumas et al. 2010, Barcelos-Silva et al. 2012

#### Chimarra (Curgia) boraceia

Flint, 1998

##### Distribution

Sao Paulo

##### Notes

Flint Jr 1998

#### Chimarra (Curgia) brasiliana

(Ulmer), 1905

##### Distribution

Parana, Santa Catarina

##### Notes

Ulmer 1905a, Almeida and Marinoni 2001

#### Chimarra (Curgia) burmeisteri

Flint, 1998

##### Distribution

Rio de Janeiro

##### Notes

Flint Jr 1998

#### Chimarra (Curgia) camposae

Flint, 1998

##### Distribution

Minas Gerais

##### Notes

Flint Jr 1998

#### Chimarra (Curgia) centrispina

Flint, 1998

##### Distribution

Minas Gerais

##### Notes

Flint Jr 1998

#### Chimarra (Curgia) cipoensis

Flint, 1998

##### Distribution

Minas Gerais

##### Notes

Flint Jr 1998

#### Chimarra (Curgia) conica

Flint, 1983

##### Distribution

**Espirito Santo**, Ceara, Goias, Minas Gerais, Mato Grosso, Rio de Janeiro, Rondonia, Santa Catarina

##### Notes

Flint Jr 1983a, Flint Jr 1998, Blahnik et al. 2004, Barcelos-Silva et al. 2012

#### Chimarra (Curgia) cultellata

Flint, 1983

##### Distribution

Distrito Federal, Minas Gerais, **Para**, **Rio de Janeiro**, Rondonia, Santa Catarina

##### Notes

Flint Jr 1983a, Flint Jr 1998, Dumas et al. 2009, Dumas et al. 2010

#### Chimarra (Curgia) donamariae

Denning & Sykora, 1968

##### Distribution

Para

##### Notes

Denning and Sykora 1968

#### Chimarra (Curgia) fittkaui

Flint, 1971

##### Distribution

Amazonas

##### Notes

Flint Jr 1971

#### Chimarra (Curgia) froehlichi

Flint, 1998

##### Distribution

Espirito Santo, Minas Gerais, Rio de Janeiro, Sao Paulo

##### Notes

Flint Jr 1998, Blahnik et al. 2004

#### Chimarra (Curgia) hyoeides

Flint, 1983

##### Distribution

**Espirito Santo**, Para, **Pernambuco**, Santa Catarina, Sao Paulo

##### Notes

Flint Jr 1983a, Flint Jr 1998, Barcelos-Silva et al. 2012, Souza et al. 2013a

#### Chimarra (Curgia) jugescens

Flint, 1998

##### Distribution

**Amazonas**

##### Notes

Flint Jr 1998, Santos and Nessimian 2009d

#### Chimarra (Curgia) medioloba

Flint, 1971

##### Distribution

Amazonas

##### Notes

Flint Jr 1971

#### Chimarra (Curgia) morio

Burmeister, 1839

##### Distribution

Bahia, Parana, Rio de Janeiro, Santa Catarina, Sao Paulo

##### Notes

Burmeister 1839, Flint Jr 1998

#### Chimarra (Curgia) parana

Flint, 1972

##### Distribution

**Espirito Santo**, Distrito Federal, Goias, Minas Gerais, **Pernambuco**, Rio deJaneiro, Santa Catarina, Sao Paulo

##### Notes

Flint Jr 1972, Flint Jr 1998, Barcelos-Silva et al. 2012, Souza et al. 2013a

#### Chimarra (Curgia) paucispina

Santos & Nessimian, 2009

##### Distribution

**Amazonas**

##### Notes

Santos and Nessimian 2009d

#### Chimarra (Curgia) petersorum

Flint, 1998

##### Distribution

Parana

##### Notes

Flint Jr 1998

#### Chimarra (Curgia) petricola

Flint, 1998

##### Distribution

**Espirito Santo**, Rio de Janeiro

##### Notes

Flint Jr 1998, Barcelos-Silva et al. 2012

#### Chimarra (Curgia) plaumanni

Flint, 1983

##### Distribution

**Espirito Santo**, Santa Catarina

##### Notes

Flint Jr 1983a, Barcelos-Silva et al. 2012

#### Chimarra (Curgia) quaternaria

Flint, 1971

##### Distribution

Amazonas

##### Notes

Flint Jr 1971

#### Chimarra (Curgia) scopuloides

Flint, 1974

##### Distribution

Goias, Para, Rondonia, Roraima, Santa Catarina

##### Notes

Flint Jr 1974a, Flint Jr 1998

#### Chimarra (Curgia) teresae

Flint, 1998

##### Distribution

**Espirito Santo**, Minas Gerais, Rio de Janeiro, Santa Catarina, Sao Paulo

##### Notes

Flint Jr 1998, Blahnik et al. 2004, Dumas et al. 2009, Barcelos-Silva et al. 2012

#### Chimarra (Curgia) tucuna

Flint, 1998

##### Distribution

Amazonas

##### Notes

Flint Jr 1998

#### Chimarra (Curgia) ypsilon

Flint, 1983

##### Distribution

Parana, Rio de Janeiro, Santa Catarina

##### Notes

Flint Jr 1983a, Almeida and Marinoni 2001

#### Chimarra (Otarrha) diaksis

Flint, 1971

##### Distribution

Amazonas

##### Notes

Flint Jr 1971

#### Chimarra (Otarrha) odonta

Blahnik, 2002

##### Distribution

**Espirito Santo**, **Minas Gerais**, Rio de Janeiro, Sao Paulo

##### Notes

Blahnik 2002, Dumas et al. 2010, Barcelos-Silva et al. 2012

#### Chimarra (undetermined) usitatissima

Flint, 1971

##### Distribution

Amazonas, Minas Gerais, Para, Rondonia

##### Notes

Flint Jr 1971, Flint Jr 1974a, Blahnik 1997

#### Dolophilodes

Ulmer, 1909

##### Notes

Ulmer 1909

#### Dolophilodes sp.

##### Distribution

**Goias**

##### Notes

Martins-Silva et al. 2008

#### Wormaldia

McLachlan, 1865

#### Wormaldia planae

Ross & King, 1956

##### Distribution

Brazil

##### Notes

Ross 1956a, Flint Jr et al. 1999b

### Polycentropodidae

#### Cernotina

Ross, 1938

##### Notes

Ross 1938, Flint Jr 1971

#### Cernotina abbreviata

Flint, 1971

##### Distribution

Para

##### Notes

Flint Jr 1971

#### Cernotina acalyptera

Flint, 1971

##### Distribution

Amazonas

##### Notes

Flint Jr 1971

#### Cernotina anhanguera

Camargos, Barcelos-Silva & Pes, 2013

##### Distribution

**Goias**

##### Notes

Barcelos-Silva et al. 2013

#### Cernotina antonina

Holzenthal & Almeida, 2003

##### Distribution

**Espirito Santo**, Minas Gerais, **Pernambuco**, Parana

##### Notes

Holzenthal and Almeida 2003, Barcelos-Silva et al. 2013, Souza et al. 2013a

#### Cernotina aruma

Santos & Nessimian, 2008

##### Distribution

**Amazonas**

##### Notes

Santos and Nessimian 2008

#### Cernotina attenuata

Flint, 1971

##### Distribution

Amazonas

##### Notes

Flint Jr 1971

#### Cernotina bibrachiata

Flint, 1971

##### Distribution

Amazonas

##### Notes

Flint Jr 1971

#### Cernotina bispicata

Camargos, Barcelos-Silva & Pes, 2013

##### Distribution

**Goias**

##### Notes

Barcelos-Silva et al. 2013

#### Cernotina cacha

Flint, 1971

##### Distribution

Amazonas, Minas Gerais

##### Notes

Flint Jr 1971, Blahnik et al. 2004

#### Cernotina cingulata

Flint, 1971

##### Distribution

Amazonas

##### Notes

Flint Jr 1971

#### Cernotina compressa

Flint, 1971

##### Distribution

Amazonas

##### Notes

Flint Jr 1971

#### Cernotina cygnea

Flint, 1971

##### Distribution

Amazonas

##### Notes

Flint Jr 1971

#### Cernotina cystophora

Flint, 1971

##### Distribution

Amazonas

##### Notes

Flint Jr 1971

#### Cernotina declinata

Flint, 1971

##### Distribution

Para

##### Notes

Flint Jr 1971

#### Cernotina decumbens

Flint, 1971

##### Distribution

Amazonas

##### Notes

Flint Jr 1971

#### Cernotina ecotura

Sykora, 1998

##### Distribution

Roraima

##### Notes

Sykora 1998

#### Cernotina encrypta

Flint, 1971

##### Distribution

Amazonas

##### Notes

Flint Jr 1971

#### Cernotina falcata

Camargos, Barcelos-Silva & Pes, 2013

##### Distribution

**Goias**

##### Notes

Barcelos-Silva et al. 2013

#### Cernotina filiformis

Flint, 1971

##### Distribution

Amazonas

##### Notes

Flint Jr 1971

#### Cernotina flexuosa

Santos & Nessimian, 2008

##### Distribution

**Amazonas**

##### Notes

Santos and Nessimian 2008

#### Cernotina lanceolata

Barcelos-Silva, Camargos & Pes, 2013

##### Distribution

**Espirito Santo**

##### Notes

Barcelos-Silva et al. 2012, Barcelos-Silva et al. 2013

#### Cernotina lazzarii

Holzenthal & Almeida, 2003

##### Distribution

Parana

##### Notes

Holzenthal and Almeida 2003

#### Cernotina lobisomem

Santos & Nessimian, 2008

##### Distribution

**Amazonas**

##### Notes

Santos and Nessimian 2008

#### Cernotina longispina

Barcelos-Silva, Camargos & Pes, 2013

##### Distribution

**Espirito Santo**

##### Notes

Barcelos-Silva et al. 2012, Barcelos-Silva et al. 2013

#### Cernotina obliqua

Flint, 1971

##### Distribution

Amazonas

##### Notes

Flint Jr 1971

#### Cernotina odonta

Santos & Nessimian, 2008

##### Distribution

**Amazonas**

##### Notes

Santos and Nessimian 2008

#### Cernotina perpendicularis

Flint, 1971

##### Distribution

Amazonas, Minas Gerais, Para

##### Notes

Flint Jr 1971, Blahnik et al. 2004

#### Cernotina pesae

Santos & Nessimian, 2008

##### Distribution

**Amazonas**

##### Notes

Santos and Nessimian 2008

#### Cernotina puri

Dumas & Nessimian, 2011

##### Distribution

**Rio de Janeiro**

##### Notes

Dumas and Nessimian 2011

#### Cernotina sexspinosa

Flint, 1983

##### Distribution

Santa Catarina

##### Notes

Flint Jr 1983a

#### Cernotina sinuosa

Barcelos-Silva, Camargos & Pes, 2013

##### Distribution

**Espirito Santo**

##### Notes

Barcelos-Silva et al. 2012, Barcelos-Silva et al. 2013

#### Cernotina spinigera

Flint, 1971

##### Distribution

**Goias**, Para, Roraima

##### Notes

Flint Jr 1971, Barcelos-Silva et al. 2013

#### Cernotina spinosior

Flint, 1991

##### Distribution

Roraima

##### Notes

Flint Jr 1991

#### Cernotina subapicalis

Flint, 1971

##### Distribution

Amazonas

##### Notes

Flint Jr 1971

#### Cernotina trispina

Flint, 1971

##### Distribution

Amazonas

##### Notes

Flint Jr 1971

#### Cernotina uara

Flint, 1971

##### Distribution

Amazonas

##### Notes

Flint Jr 1971

#### Cernotina unguiculata

Flint, 1971

##### Distribution

Para

##### Notes

Flint Jr 1971

#### Cernotina verticalis

Flint, 1971

##### Distribution

Amazonas

##### Notes

Flint Jr 1971

#### Cyrnellus

Banks, 1913

##### Notes

Banks 1913, Flint Jr 1971

#### Cyrnellus arotron

Flint, 1971

##### Distribution

Amazonas, **Mato Grosso do Sul**, Para

##### Notes

Flint Jr 1971, Dumas et al. 2010

#### Cyrnellus bifidus

Flint, 1971

##### Distribution

Amazonas, **Mato Grosso do Sul**

##### Notes

Flint Jr 1971, Dumas et al. 2010

#### Cyrnellus collaris

Flint, 1971

##### Distribution

Amazonas

##### Notes

Flint Jr 1971

#### Cyrnellus fraternus

(Banks), 1905

##### Distribution

Amazonas, **Bahia**, **Espirito Santo**, Minas Gerais, **Mato Grosso**, **Mato Grosso do Sul**, Para, Parana, **Rio de Janeiro**, Santa Catarina

##### Notes

Banks 1905, Blahnik et al. 2004, Dumas et al. 2009, Dumas et al. 2010, Nogueira and Cabette 2011

#### Cyrnellus mammillatus

Flint, 1971

##### Distribution

Amazonas, Minas Gerais, **Mato Grosso do Sul**, Para, **Pernambuco**, Parana, Rio de Janeiro, Sao Paulo

##### Notes

Flint Jr 1971, Blahnik et al. 2004, Dumas et al. 2010, Souza et al. 2013a

#### Cyrnellus risi

(Ulmer), 1907

##### Distribution

Amazonas, **Espirito Santo**, Minas Gerais, Para

##### Notes

Ulmer 1907a, Blahnik et al. 2004, Barcelos-Silva et al. 2012

#### Cyrnellus ulmeri

Flint, 1971

##### Distribution

Amazonas, Para

##### Notes

Flint Jr 1971

#### Nyctiophylax

Brauer, 1865

##### Notes

Brauer 1865, Neboiss 1993

#### Nyctiophylax neotropicalis

Flint, 1971

##### Distribution

Amazonas, **Espirito Santo**, Minas Gerais, **Para**, **Pernambuco**, Parana, Rio de Janeiro

##### Notes

Flint Jr 1971, Blahnik et al. 2004, Dumas et al. 2010, Barcelos-Silva et al. 2012, Souza et al. 2013a

#### Polycentropus

Curtis, 1835

#### Polycentropus acinaciformis

Hamilton & Holzenthal, 2011

##### Distribution

**Minas Gerais**

##### Notes

Hamilton and Holzenthal 2011

#### Polycentropus amphirhamphus

Hamilton & Holzenthal, 2011

##### Distribution

**Riode Janeiro**, **Santa Catarina**, **Sao Paulo**

##### Notes

Hamilton and Holzenthal 2011

#### Polycentropus ancistrus

Hamilton & Holzenthal, 2011

##### Distribution

**Sao Paulo**

##### Notes

Hamilton and Holzenthal 2011

#### Polycentropus boraceia

Hamilton & Holzenthal, 2011

##### Distribution

**Sao Paulo**

##### Notes

Hamilton and Holzenthal 2011

#### Polycentropus caaete

Hamilton & Holzenthal, 2011

##### Distribution

**Parana**, **Santa Catarina**, **Sao Paulo**

##### Notes

Hamilton and Holzenthal 2011

#### Polycentropus cachoeira

Hamilton & Holzenthal, 2011

##### Distribution

**Santa Catarina**

##### Notes

Hamilton and Holzenthal 2011

#### Polycentropus carioca

Hamilton & Holzenthal, 2011

##### Distribution

**Rio de Janeiro**

##### Notes

Hamilton and Holzenthal 2011

#### Polycentropus carolae

Hamilton & Holzenthal, 2011

##### Distribution

**Rio de Janeiro**

##### Notes

Hamilton and Holzenthal 2011

#### Polycentropus cipoensis

Hamilton & Holzenthal, 2011

##### Distribution

**Minas Gerais**, **Sao Paulo**

##### Notes

Hamilton and Holzenthal 2011

#### Polycentropus cheliceratus

Hamilton & Holzenthal, 2011

##### Distribution

**Rio de Janeiro**

##### Notes

Hamilton and Holzenthal 2011

#### Polycentropus fluminensis

Hamilton & Holzenthal, 2011

##### Distribution

**Espirito Santo**, **Minas Gerais**, **Rio de Janeiro**

##### Notes

Hamilton and Holzenthal 2011, Barcelos-Silva et al. 2012

#### Polycentropus froehlichi

Hamilton & Holzenthal, 2011

##### Distribution

**Santa Catarina**

##### Notes

Hamilton and Holzenthal 2011

#### Polycentropus galharada

Hamilton & Holzenthal, 2011

##### Distribution

**Sao Paulo**

##### Notes

Hamilton and Holzenthal 2011

#### Polycentropus graciosa

Hamilton & Holzenthal, 2011

##### Distribution

**Parana**

##### Notes

Hamilton and Holzenthal 2011

#### Polycentropus inusitatus

Hamilton & Holzenthal, 2011

##### Distribution

**Minas Gerais**, **Rio de Janeiro**

##### Notes

Hamilton and Holzenthal 2011, Dumas and Nessimian 2012

#### Polycentropus itatiaia

Hamilton & Holzenthal, 2011

##### Distribution

**Minas Gerais**, **Rio de Janeiro**

##### Notes

Hamilton and Holzenthal 2011

#### Polycentropus minero

Hamilton & Holzenthal, 2011

##### Distribution

**Minas Gerais**

##### Notes

Hamilton and Holzenthal 2011

#### Polycentropus paprockii

Hamilton & Holzenthal, 2011

##### Distribution

**Minas Gerais**

##### Notes

Hamilton and Holzenthal 2011

#### Polycentropus rosalysae

Hamilton & Holzenthal, 2011

##### Distribution

**Minas Gerais**, **Rio de Janeiro**, **Sao Paulo**

##### Notes

Hamilton and Holzenthal 2011, Dumas and Nessimian 2012

#### Polycentropus santateresae

Hamilton & Holzenthal, 2011

##### Distribution

**Espirito Santo**, **Minas Gerais**

##### Notes

Hamilton and Holzenthal 2011

#### Polycentropus soniae

Hamilton & Holzenthal, 2011

##### Distribution

**Parana**

##### Notes

Hamilton and Holzenthal 2011

#### Polycentropus tripui

Hamilton & Holzenthal, 2011

##### Distribution

**Minas Gerais**

##### Notes

Hamilton and Holzenthal 2011

#### Polycentropus urubici

Holzenthal & Almeida, 2003

##### Distribution

**Minas Gerais**, Parana, Santa Catarina

##### Notes

Holzenthal and Almeida 2003, Dumas et al. 2010

#### Polycentropus verruculus

Hamilton & Holzenthal, 2011

##### Distribution

**Minas Gerais**, **Sao Paulo**

##### Notes

Hamilton and Holzenthal 2011

#### Polycentropus virginiae

Hamilton & Holzenthal, 2011

##### Distribution

**Minas Gerais**

##### Notes

Hamilton and Holzenthal 2011

#### Polyplectropus

Ulmer, 1905

##### Notes

Ulmer 1905a, Flint Jr 1968, Bueno-Soria 1990, Chamorro and Holzenthal 2010

#### Polyplectropus alatespinus

Chamorro & Holzenthal, 2010

##### Distribution

**Minas Gerais**, **Rio de Janeiro**, **Sao Paulo**

##### Notes

Chamorro and Holzenthal 2010

#### Polyplectropus alleni

(Yamamoto), 1967

##### Distribution

Minas Gerais

##### Notes

Yamamoto 1967, Blahnik et al. 2004, Chamorro and Holzenthal 2010

#### Polyplectropus annulicornis

Ulmer, 1905

##### Distribution

**Parana**, **Rio de Janeiro**, Rio Grande doSul, **Santa Catarina**

##### Notes

Ulmer 1905b, Chamorro and Holzenthal 2010, Dumas and Nessimian 2012

#### Polyplectropus banksianus

Flint, 1971

##### Distribution

Amazonas

##### Notes

Flint Jr 1971, Chamorro and Holzenthal 2010

#### Polyplectropus brachyscolus

Flint, 1971

##### Distribution

Amazonas

##### Notes

Flint Jr 1971, Chamorro and Holzenthal 2010

#### Polyplectropus brasilensis

Chamorro & Holzenthal, 2010

##### Distribution

**Rio de Janeiro**, **Sao Paulo**

##### Notes

Chamorro and Holzenthal 2010

#### Polyplectropus dubitatus

Flint, 1983

##### Distribution

**Minas Gerais**, **Parana**, Santa Catarina

##### Notes

Flint Jr 1983a, Chamorro and Holzenthal 2010

#### Polyplectropus elongatus

(Yamamoto), 1966

##### Distribution

**Minas Gerais**

##### Notes

Yamamoto 1966, Chamorro and Holzenthal 2010

#### Polyplectropus flavicornis

Ulmer, 1905

##### Distribution

**Minas Gerais**, Santa Catarina

##### Notes

Ulmer 1905a, Chamorro and Holzenthal 2010

#### Polyplectropus fuscatus

Flint, 1983

##### Distribution

Santa Catarina

##### Notes

Flint Jr 1983a, Chamorro and Holzenthal 2010

#### Polyplectropus gaesum

Chamorro & Holzenthal, 2010

##### Distribution

**Minas Gerais**

##### Notes

Chamorro and Holzenthal 2010

#### Polyplectropus hollyae

Chamorro & Holzenthal, 2010

##### Distribution

**Minas Gerais**

##### Notes

Chamorro and Holzenthal 2010

#### Polyplectropus hystricosus

Chamorro & Holzenthal, 2010

##### Distribution

**Minas Gerais**, **Rio de Janeiro**

##### Notes

Chamorro and Holzenthal 2010, Dumas and Nessimian 2012

#### Polyplectropus inarmatus

Flint, 1971

##### Distribution

Amazonas

##### Notes

Flint Jr 1971, Chamorro and Holzenthal 2010

#### Polyplectropus juliae

Chamorro & Holzenthal, 2010

##### Distribution

**Minas Gerais**

##### Notes

Chamorro and Holzenthal 2010

#### Polyplectropus matatlanticus

Chamorro & Holzenthal, 2010

##### Distribution

**Minas Gerais**, **Sao Paulo**

##### Notes

Chamorro and Holzenthal 2010

#### Polyplectropus minensium

Chamorro & Holzenthal, 2010

##### Distribution

**Minas Gerais**

##### Notes

Chamorro and Holzenthal 2010

#### Polyplectropus novafriburgensis

Chamorro & Holzenthal, 2010

##### Distribution

**Rio de Janeiro**

##### Notes

Chamorro and Holzenthal 2010

#### Polyplectropus petrae

Chamorro & Holzenthal, 2010

##### Distribution

**Minas Gerais**

##### Notes

Chamorro and Holzenthal 2010

#### Polyplectropus pratherae

Chamorro & Holzenthal, 2010

##### Distribution

**Minas Gerais**

##### Notes

Chamorro and Holzenthal 2010

#### Polyplectropus rodmani

Chamorro & Holzenthal, 2010

##### Distribution

**Sao Paulo**

##### Notes

Chamorro and Holzenthal 2010

#### Polyplectropus rondoniensis

Chamorro & Holzenthal, 2010

##### Distribution

**Rondonia**

##### Notes

Chamorro and Holzenthal 2010

#### Polyplectropus profaupar

Holzenthal & Almeida, 2003

##### Distribution

**Minas Gerais**, Parana, **Rio de Janeiro**, Santa Catarina

##### Notes

Holzenthal and Almeida 2003, Dumas et al. 2009, Chamorro and Holzenthal 2010, Dumas et al. 2010

#### Polyplectropus spiculifer

Flint, 1971

##### Distribution

Amazonas

##### Notes

Flint Jr 1971, Chamorro and Holzenthal 2010

#### Polyplectropus tragularius

Chamorro & Holzenthal, 2010

##### Distribution

**Espirito Santo**, **Minas Gerais**, **Sao Paulo**

##### Notes

Chamorro and Holzenthal 2010

#### Polyplectropus ulmeriana

Flint, 1983

##### Distribution

Santa Catarina

##### Notes

Flint Jr 1983a, Chamorro and Holzenthal 2010

### Sericostomatidae

#### Grumicha

Müller, 1879

##### Notes

Müller 1879a, Flint Jr et al. 1999a

#### Grumicha grumicha

(Vallot), 1855

##### Distribution

Santa Catarina, Sao Paulo

##### Notes

Vallot 1855, Blahnik et al. 2004

#### Notidobiella

Schmid, 1955

##### Notes

Schmid 1955

#### Notidobiella amazoniana

Holzenthal & Blahnik, 2010

##### Distribution

**Amazonas**

##### Notes

Holzenthal and Blahnik 2010

#### Notidobiella brasiliana

Holzenthal & Blahnik, 2010

##### Distribution

**Sao Paulo**

##### Notes

Holzenthal and Blahnik 2010

### Xiphocentronidae

#### Machairocentron

Schmid, 1982

##### Notes

Schmid 1982

#### Machairocentron falciforme

Pes & Hamada, 2013

##### Distribution

**Amazonas**

##### Notes

Pes et al. 2013

#### Machairocentron sp.

##### Distribution

**Amazonas**

##### Notes

Schmid 1982, Pes et al. 2005

#### Xiphocentron

Brauer, 1870

##### Notes

Brauer 1870, Schmid 1982

#### Xiphocentron (Antillotrichia) ilionea

Schmid, 1982

##### Distribution

**Rio de Janeiro**, Sao Paulo

##### Notes

Schmid 1982, Dumas et al. 2009

#### Xiphocentron (Antillotrichia) sclerothrix

Pes & Hamada, 2013

##### Distribution

**Amazonas**, **Amapa**

##### Notes

Pes et al. 2013

#### Xiphocentron (Antillotrichia) steffeni

(Marlier), 1964

##### Distribution

**Minas Gerais**, **Rio de Janeiro**, Sao Paulo

##### Notes

Marlier 1964a, Dumas et al. 2010

#### Xiphocentron (undetermined) saltuum

Müller, 1921

##### Distribution

Brazil

##### Notes

Müller 1921

## Discussion

**Figure 1.**
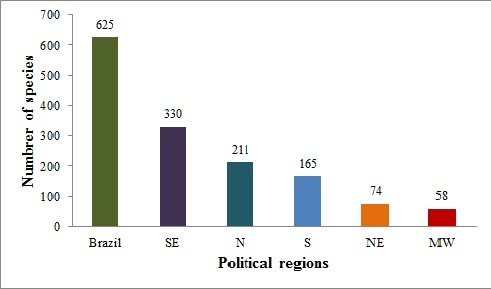
Number of species by political regions. SE (Southeast), N (North), S (South), NE (Northeast), MW (Midwest). Chart based on data in Suppl. material 1.

The number of Trichoptera species recorded from Brazil until September, 2014 is 625. This number represents a 65.34% increase in species records since 2004. Ninety percent of the new records were also new species to science. The number of new records since the first Brazilian Checklist is 252, including 223 new species and 29 new country records. The Hydropsychidae rank first in species richness totaling 124 species, followed by Hydroptilidae with 102 species and Polycentropodidae with 97 species. Despite of their ubiquity, caddisflies remain unrecorded in 4 Brazilian states (AL, MA, RN, TO). There is a clear relation between the concentration of universites and researchers and the number of species recorded for a region. Southeastern Brazil, home of the most and the largest universities in Brazil, ranks first with 330 species recorded followed by the Northern region (211 species) and Southern region (165 species). The regions with the greatest lack of knowledge, and which should be considered priorities for Trichoptera inventories, are the Northeast with 74 species and Midwest with 58 species (Fig. 1).

The number of regional inventories published since the first Brazilian Trichoptera Checklist is 10 (Blahnik et al. 2004, Ribeiro et al. 2009, Dumas et al. 2009, Nogueira and Cabette 2011, Calor 2011, Dumas and Nessimian 2012, Barcelos-Silva et al. 2012, Souza et al. 2013a, Costa et al. 2014, Quinteiro et al. 2014). It is important to increase the number of regional surveys and publication of regional checklists. The benefits of regional surveys associated with georeferenced data made available online are one of the most important advancements to comprehensive biodiversity data studies (Chavan and Penev 2011). Primary data on Trichoptera biodiversity should be made freely available through inititives like the Global Biodiversity Information System (http://www.gbif.org/). Brazil has undertaken a major initiative of making scientific collections biodiversity information available online through the *Centro de Referências em Informação Ambiental* – CRIA (www.cria.org.br). The numbers acquired by CRIA (August 2014) are impressive and include data from 358 scientific collections, 5,385,443 georreferenced distribution records, and 447,657 species names. The number of Trichoptera records retrieved from CRIA was 6,404. The linkage between scientific journals and such databases should be greatly improved to achieve open access to biodiversity data.

**Figure 2.**
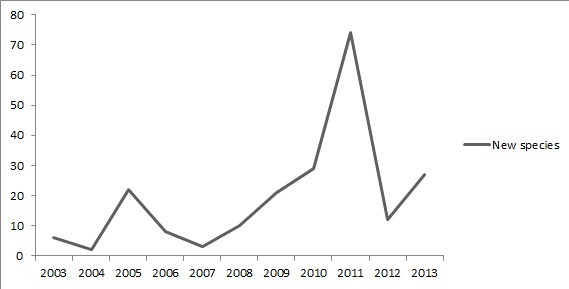
Number of described species per year since 2003 for Brazil. Chart based on data in Suppl. material 2.

Over six hundred species of Trichoptera have been described for Brazil since the first species was recorded in 1833 (*Macrostemum maculatum* (Perty) 1833. An average of 2.21 species/year were described between 1833 and the publications of the first comprehensive checklist (Paprocki et al. 2004). Since 2004 the rate of species description has increased dramatically, with an average of 20.2 species/year (Fig. 2).

## Supplementary Material

Supplementary material 1 Figure 1 source Data type: spreadsheet, chart
File: oo_8328.xlsx Paprocki, H., França, D.

Supplementary material 2 Figure 2 source Data type: spreadsheet, chart
File: oo_8329.xlsx Paprocki, H., França, D.
